# Prevalence of Perinatal Anxiety and Related Disorders in Low- and Middle-Income Countries: A Systematic Review and Meta-Analysis

**DOI:** 10.1001/jamanetworkopen.2023.43711

**Published:** 2023-11-17

**Authors:** Alexandra Roddy Mitchell, Hannah Gordon, Jessica Atkinson, Anthea Lindquist, Susan P. Walker, Anna Middleton, Stephen Tong, Roxanne Hastie

**Affiliations:** 1 Mercy Perinatal, Department of Obstetrics and Gynaecology, The University of Melbourne, Heidelberg, Victoria, Australia; 2 Mercy Hospital for Women, Heidelberg, Victoria, Australia

## Abstract

**Importance:**

Anxiety disorders are associated with poor maternal and neonatal outcomes. Women in low- and middle-income countries (LMICs) are thought to be disproportionally burdened by these disorders, yet their prevalence is unclear.

**Objective:**

To conduct a systematic review and meta-analysis to determine the prevalence of 6 anxiety and related disorders among perinatal women in LMICs.

**Data Sources:**

Embase, MEDLINE, PsycINFO, Cochrane Library, CINAHL, and Web of Science databases were searched from inception until September 7, 2023.

**Study Selection:**

Studies conducted in World Bank–defined LMICs and reporting prevalence of generalized anxiety disorder, obsessive-compulsive disorder, social anxiety disorder, posttraumatic stress disorder, panic disorder, or adjustment disorder during the perinatal period (conception to 12 months post partum) using a validated method were included.

**Data Extraction and Synthesis:**

This study followed the Preferred Reporting Items for Systematic Reviews and Meta-Analyses reporting guideline. Study eligibility, extracted data, and risk of bias of included studies were assessed by 2 independent reviewers. Random-effects meta-analysis was used to estimate pooled point prevalence. Subgroup analyses were performed by specific anxiety disorder.

**Main Outcomes and Measures:**

Main outcomes were prevalence estimates of each anxiety disorder, measured as percentage point estimates and corresponding 95% CIs.

**Results:**

At total of 10 617 studies were identified, 203 of which met the inclusion criteria and reported the outcomes of 212 318 women from 33 LMICs. Generalized anxiety disorder was the most reported (184 studies [90.6%]) and most prevalent disorder at 22.2% (95% CI, 19.4%-25.0%; n = 173 553). Posttraumatic stress disorder was the second most prevalent (8.3%; 95% CI, 5.0%-12.2%; 33 studies; n = 22 452). Adjustment disorder was least prevalent (2.9%; 95% CI, 0.0%-14.1%; 2 studies; n = 475). The prevalence of generalized anxiety varied by country income status, with the highest prevalence among lower-middle–income countries (27.6%; 95% CI, 21.6%-33.9%; 59 studies; n = 25 109), followed by low-income (24.0%; 95% CI, 15.3%-33.8%; 11 studies; n = 4961) and upper-middle–income (19.1%; 95% CI, 16.0%-22.4%; 110 studies; n = 138 496) countries.

**Conclusions and Relevance:**

These findings suggest that 1 in 5 women living in LMICs experience anxiety disorders during pregnancy and post partum. Targeted action is needed to reduce this high burden.

## Key Points

**Question:**

What is the prevalence of anxiety and related disorders during pregnancy and in the year following birth among women living in low- and middle-income countries?

**Findings:**

In this systematic review and meta-analysis of 203 studies including 212 318 women, 1 in 5 pregnant and postpartum women living in low- and middle-income counties were found to experience generalized anxiety disorder. Additionally, 8.3% of women experienced posttraumatic stress disorder.

**Meaning:**

The findings suggest that addressing anxiety and related disorders during pregnancy and in the year following birth may improve outcomes for women and babies.

## Teaser

This systematic review and meta-analysis examines the prevalence of anxiety and related disorders among perinatal women living in low- and middle-income countries.

## Introduction

In many high-income countries, anxiety disorders are the most prevalent mental health disorders.^1^ They can arise more often during the perinatal period (from conception until 12 months post childbirth) than at other times in a woman’s life.^2^ Furthermore, perinatal anxiety and related disorders are associated with adverse outcomes for women and their babies.^3^ For the offspring, adverse outcomes include preterm birth, poor neurocognitive development, and longer-term outcomes, such as increased risk of cardiovascular disease and mental illness in adulthood.^4,5^ For women, perinatal anxiety disorders are associated with substance use disorders, recurrent mental illness, and suicide.^6,7,8^ Anxiety and related disorders (generalized anxiety disorder, obsessive-compulsive disorder, social anxiety disorder, posttraumatic stress disorder, panic disorder, and adjustment disorder) are associated with a high burden of disease and economic cost.

Many predisposing factors for anxiety disorders, including poverty, gender inequity, and intimate partner violence, are common in low- and middle-income countries (LMICs).^9,10^ These factors, combined with reduced availability and access to mental health care and treatment, often mean that women living in LMICs are at higher risk of developing perinatal anxiety disorders and the associated sequelae.^11^ Thus, an increased focus on perinatal anxiety disorders in LMICs is justified.

Although perinatal anxiety disorders have received growing attention in many high-income countries, this has not been the case among LMICs.^4^ Global prevalence of perinatal anxiety is estimated at 15% to 23%^6,12^; however, it has been suggested that the burden of disease is greater for LMICs compared with high-income countries.^13^ While thought to be common, the prevalence of these disorders in LMICs is unclear.^13^ Establishing prevalence estimates is a key first step in understanding the burden experienced by women in LMICs and addressing this important public health issue. This study aims to estimate the prevalence of perinatal anxiety and related disorders among women living in LMICs.

## Methods

### Search Strategy and Selection Criteria

We conducted a systematic review and meta-analysis to determine the prevalence of anxiety and related disorders among perinatal women in LMICs. The study was registered with PROSPERO (CRD42021242901) and reported according to the Preferred Reporting Items for Systematic Reviews and Meta-Analyses (PRISMA) reporting guideline.^14^ Ethical approval was not required for this study as it only uses data from studies that are already published.

We searched the MEDLINE, PsycINFO, Embase, Cochrane Library, CINAHL, and Web of Science databases from inception until September 7, 2023 (eMethods 1 in Supplement 1). The primary search included terms related to mental health disorders, prevalence, and the perinatal period (defined as any time during pregnancy and up to 12 months after birth) and was restricted to studies performed in countries defined by the World Bank as low, lower-middle, or upper-middle income (eMethods 2 in Supplement 1). Studies were included if they measured the prevalence of a mental health disorder in perinatal women using a validated method. We deemed a validated method as a diagnostic interview or a screening tool that had previously been tested and validated. Cohort studies, cross-sectional studies, baseline data from randomized clinical trials, and prevalence data from control participants in case-control studies were included.

We excluded studies conducted in high-income countries, studies not published in English, case studies, editorials, review articles, and guidelines. In this review, we included studies that reported the prevalence of disorders according to the *Diagnostic and Statistical Manual of Mental Disorders* (Fifth Edition) (*DSM-5*),^15^ including generalized anxiety disorder, obsessive-compulsive disorder, social anxiety disorder, posttraumatic stress disorder, panic disorder, and adjustment disorder. Studies that only used the term *anxiety* were included in a generalized anxiety category. Studies measuring conditions not included in the *DSM-5*, such as pregnancy-related anxiety, were not included. Studies that reported the combined prevalence of anxiety and depression were also excluded.

References identified from the systematic search were uploaded into Covidence systematic review software.^16^ Duplicate references were removed, and 2 reviewers (A.R.M. [midwife, PhD candidate] and H.G. [obstetric registrar, PhD candidate], A.M. [midwife], or J.A. [PhD candidate]) independently assessed titles and abstracts, screened full texts, and extracted data from eligible studies. Discrepancies were resolved in consultation with a third reviewer (R.H. [perinatal epidemiologist]).

The following data were extracted: author, year of publication, country of study, country income status, total number of participants, type of disorder, number of participants with disorder, study design, setting, method of assessment (ie, self-assessed screening tool vs diagnostic interview), assessment tool, and timing of assessment (antenatal, postnatal, or combined). We further extracted the following data on maternal characteristics identified a priori as placing women at increased risk of experiencing a mental health disorder: adolescence,^4,8^ experience of intimate partner violence,^3,17^ HIV positivity,^18^ and current experience of war or conflict^19^ or the COVID-19 pandemic.^20^

### Statistical Analysis

Prevalence data of each disorder were extracted as raw proportions. Random-effects meta-analysis was used with the Freeman-Tukey double-arcsine transformation to generate point estimates and 95% CIs.^21^ First, we calculated pooled point estimates of prevalence by type of anxiety disorder. Next, among studies reporting the prevalence of generalized anxiety, we performed prespecified subgroup analyses by (1) World Bank–defined income status, (2) World Bank–defined region, (3) timing of assessment, (4) method of assessment, (5) study setting, (6) study design, and (7) maternal risk factors identified a priori (adolescence, HIV status, intimate partner violence, and current experience of war or the COVID-19 pandemic). Where possible (≥2 studies), random-effects meta-analysis was performed to assess within-group differences and presented as pooled point estimates and corresponding 95% CIs and *P* values.

For longitudinal studies reporting several prevalence estimates over time, to avoid double counting of participants, only 1 estimate was extracted. This estimate was closest to birth (excluding the first 2 weeks post partum to allow for a period of “baby blues”).^22^ Where both antenatal and postnatal estimates were reported, the antenatal estimate was selected for the overall analysis. Point estimates of proportions and corresponding 95% CIs were calculated and displayed in forest plots. For pooled estimates, τ^2^ was used to estimate the between-study variance, and the *I*^2^ statistic was used to quantify heterogeneity.^23^

Risk of bias was assessed independently by 2 reviewers (A.R.M. and H.G., A.M., or J.A.) using a modified version of the Newcastle-Ottawa Scale (eMethods 3 and eTable 1 in Supplement 1)^24^ across 3 domains (selection of participants, comparability of groups, and ascertainment of outcome) and given a score out of 7. Discrepancies were resolved by a third reviewer (R.H.).

We performed sensitivity analyses first by excluding studies deemed at high risk of bias, as suggested by Higgins et al,^25^ and omitted studies scoring less than 5 on the modified Newcastle-Ottawa Scale. We further used subgroup analysis to investigate heterogeneity. All statistical tests were 2-sided, and a *P* < .05 was considered statistically significant. Stata/MP, version 18 (StataCorp LLC), was used for the analysis.^26^

## Results

**Figure 1.**
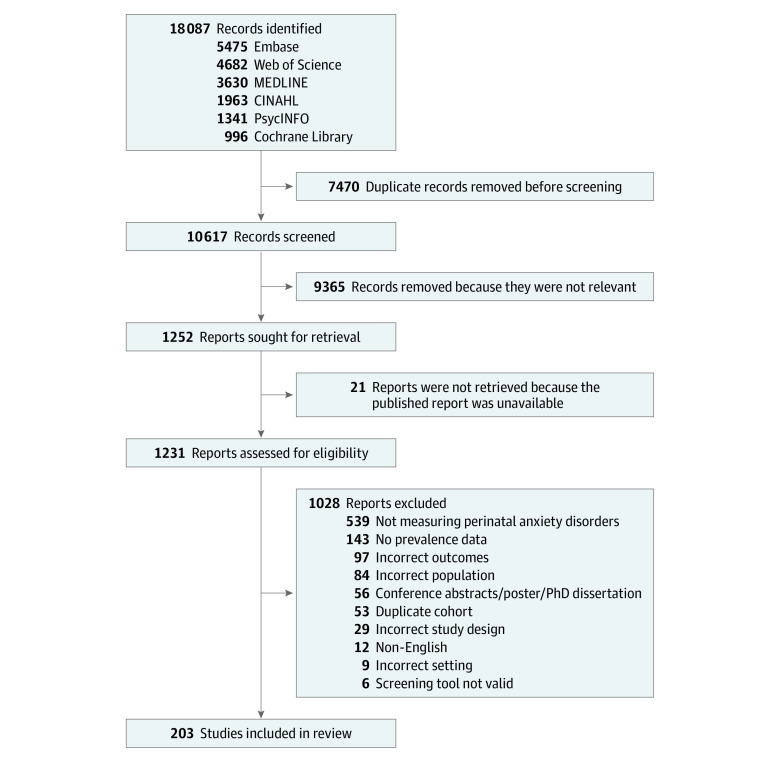
PRISMA Flow Diagram

Our search identified 10 617 studies; 1231 full texts were reviewed, and of these, 203 studies were included (Figure 1; eTable 2 in Supplement 1).^27,28,29,30,31,32,33,34,35,36,37,38,39,40,41,42,43,44,45,46,47,48,49,50,51,52,53,54,55,56,57,58,59,60,61,62,63,64,65,66,67,68,69,70,71,72,73,74,75,76,77,78,79,80,81,82,83,84,85,86,87,88,89,90,91,92,93,94,95,96,97,98,99,100,101,102,103,104,105,106,107,108,109,110,111,112,113,114,115,116,117,118,119,120,121,122,123,124,125,126,127,128,129,130,131,132,133,134,135,136,137,138,139,140,141,142,143,144,145,146,147,148,149,150,151,152,153,154,155,156,157,158,159,160,161,162,163,164,165,166,167,168,169,170,171,172,173,174,175,176,177,178,179,180,181,182,183,184,185,186,187,188,189,190,191,192,193,194,195,196,197,198,199,200,201,202,203,204,205,206,207,208,209,210,211,212,213,214,215,216,217,218,219,220,221,222,223,224,225,226,227,228,229^ Cumulatively, outcomes were reported for 212 318 perinatal women (from conception to 12 months post birth) from 33 LMICs. The prevalence estimates of 6 anxiety and related disorders were reported: generalized anxiety disorder, social anxiety disorder, posttraumatic stress disorder, obsessive-compulsive disorder, panic disorder, and adjustment disorder.

**Figure 2.**
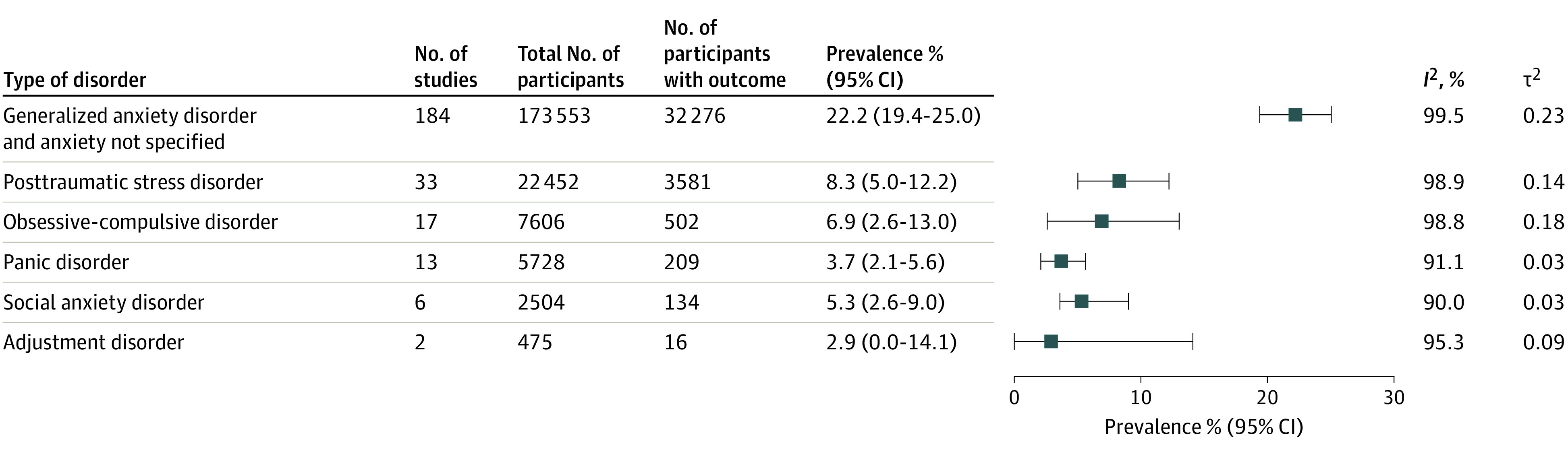
Prevalence of Anxiety and Related Disorders in Low- and Middle-Income Countries

Generalized anxiety disorder (including anxiety not specified) was the most commonly reported anxiety disorder, examined by 184 of 203 studies (90.6%).^27,28,29,30,31,32,33,34,35,36,37,38,39,40,41,42,43,44,45,46,47,48,49,50,51,52,53,54,55,56,57,59,60,61,62,63,64,65,66,67,68,69,70,71,72,73,74,75,76,77,78,79,80,81,82,83,84,85,86,87,88,89,90,91,92,93,94,95,96,97,98,99,100,101,102,103,104,105,106,107,108,109,110,111,112,113,116,117,118,119,120,121,122,123,124,125,126,127,128,129,130,131,132,133,134,135,136,137,138,139,140,141,142,143,144,145,146,147,148,149,150,151,152,153,154,155,156,157,158,159,160,161,162,163,164,165,167,168,169,170,171,172,173,174,175,176,177,178,179,180,181,182,183,184,185,186,187,188,189,190,191,192,193,195,196,197,198,199,201,203,204,205,209,211,212,213,214,217,218,219,220,221,222,223,224,226,227,228,229^ The pooled point prevalence for generalized anxiety disorder was 22.2% (95% CI, 19.4%-25.0%; n = 173 553). The next most common disorder was posttraumatic stress disorder (33 studies [16.3%]^47,48,49,50,53,54,56,57,60,98,106,114,115,116,117,119,122,130,156,157,158,184,198,199,200,206,207,208,210,213,214,215,216,225,227^), with a prevalence of 8.3% (95% CI, 5.0%-12.2%; n = 22 452) of women. Obsessive-compulsive disorder was assessed in 17 studies^44,47,48,49,50,53,54,57,58,89,130,141,143,184,202,206,224^ and had a pooled point prevalence of 6.9% (95% CI, 2.6%-13.0%; n = 7606). Six studies^49,54,57,166,184,194^ investigated social anxiety disorder, with a pooled prevalence of 5.3% (95% CI, 2.6%-9.0%; n = 2504). Panic disorder had a prevalence of 3.7% (95% CI, 2.1%-5.6%; n = 5728; 13 studies^47,49,50,53,54,57,130,138,166,169,184,194,229^). Only 2 studies^60,143^ measured the prevalence of adjustment disorder (2.9%; 95% CI, 0.0%-14.1%]; n = 475) (Figure 2; eFigures 1-6 in Supplement 1).

**Figure 3.**
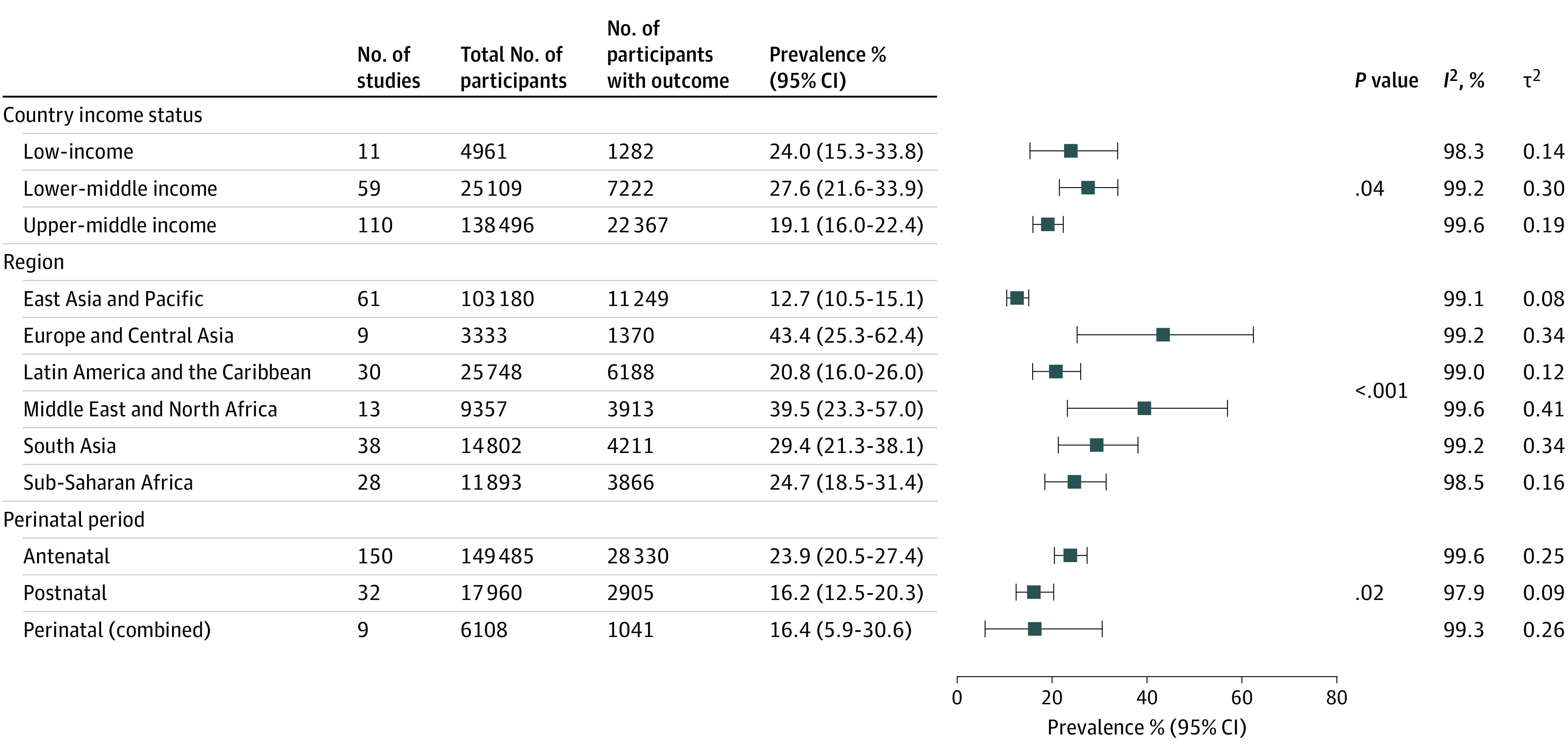
Generalized Anxiety Disorder Subgroup Analysis

Given that generalized anxiety disorder was reported across 184 studies, we were able to further interrogate the prevalence of this disorder across important subgroups (Figure 3). While investigating generalized anxiety by country income status, we found a higher prevalence among studies performed in lower-middle–income countries (27.6%; 95% CI, 21.6%-33.9%; 59 studies^27,29,30,31,32,60,76,119,120,121,129,130,131,132,133,134,135,136,137,138,139,140,141,142,143,144,145,146,168,174,175,176,177,178,179,180,181,182,183,184,185,186,187,188,189,190,191,192,193,195,196,197,209,211,212,213,214,228,229^; n = 25 109) compared with low-income countries (24.0%; 95% CI, 15.3%-33.8%; 11 studies^28,122,123,124,125,126,127,128,169,199,227^; n = 4961). Upper-middle–income countries had the lowest prevalence (19.1%; 95% CI, 16.0%-22.4%; 110 studies^33,34,35,36,37,38,39,40,41,42,43,44,45,46,47,48,49,50,51,52,53,54,55,56,57,59,61,62,63,64,65,66,67,68,69,70,71,72,73,74,75,77,78,79,80,81,82,83,84,85,86,87,88,89,90,91,92,93,94,95,96,97,99,100,101,102,103,104,105,106,107,108,109,110,111,112,113,116,117,118,147,148,149,150,151,152,153,154,155,159,160,161,162,163,164,165,167,198,201,203,204,205,217,218,219,220,221,222,223,224,226^; n = 138 496).

While investigating generalized anxiety by region, we found significant variation (*P* < .001). Europe and Central Asia had the highest prevalence (43.4%; 95% CI, 25.3%-62.4%; n = 3333). This subgroup included only 9 studies,^217,218,219,220,221,222,223,224,226^ all of which were performed in Turkey. The lowest prevalence was found in East Asia and the Pacific (12.7%; 95% CI, 10.5%-15.1%; 61 studies^61,62,63,64,65,66,67,68,69,70,71,72,73,74,75,77,78,79,80,81,82,83,84,85,86,87,88,89,90,91,92,93,94,95,96,97,99,100,101,102,103,104,105,106,107,108,109,110,111,112,113,116,147,148,161,162,163,164,165,228,229^; n = 103 180). Of the studies conducted in East Asia and the Pacific, 53 (86.9%) were conducted in China^61,62,63,64,65,66,67,68,69,70,71,72,73,74,75,76,77,78,79,80,81,82,83,84,85,86,87,88,89,90,91,92,93,94,95,96,97,99,100,101,102,103,104,105,106,107,108,109,110,111,112,113,116^ (eFigure 7 in Supplement 1).

The majority of studies reported the prevalence of generalized anxiety antenatally (150 [81.5%]^27,28,29,31,33,35,36,37,39,40,41,42,43,44,45,46,47,48,49,50,51,52,53,55,57,59,61,62,63,64,66,67,70,71,72,73,74,75,76,77,78,79,80,81,82,83,84,85,86,87,88,89,90,91,92,94,95,96,97,99,101,103,104,106,108,109,110,111,112,113,116,117,119,121,123,124,125,126,127,129,131,132,135,136,137,138,140,141,142,144,145,146,147,148,149,150,151,152,154,159,160,161,163,164,165,167,170,172,173,174,175,177,178,179,180,181,184,185,187,188,189,190,191,192,193,195,196,197,198,199,201,203,204,205,209,210,211,212,213,214,217,218,219,220,221,222,223,224,226,228^). Among these, the pooled point prevalence was 23.9% (95% CI, 20.5%-27.4%; n = 149 485). The prevalence was significantly lower post partum (16.2%; 95% CI, 12.5%-20.3%; 32 studies^30,32,34,38,54,65,68,69,80,86,90,93,100,107,118,120,122,130,133,139,153,155,168,170,175,176,182,183,186,199,227,229^; n = 17 960; *P* = .02). Nine studies^60,102,105,128,134,143,162,169,171^ measured the prevalence of pregnant and postpartum women, with combined results showing a prevalence of 16.4% (95% CI, 5.9%-30.6%; n = 6108).

Next, we assessed the prevalence of generalized anxiety by method of assessment. Screening tools were used in 153 studies^28,29,30,31,32,33,34,38,39,41,45,46,51,52,59,60,61,62,63,64,65,66,67,68,69,70,71,72,73,74,75,76,77,78,79,80,81,82,83,84,85,86,87,88,89,90,91,92,93,94,95,96,97,99,100,101,102,103,104,105,106,107,108,109,110,111,112,113,116,117,118,119,120,121,122,123,124,125,126,127,128,129,131,132,133,134,135,136,137,139,140,142,144,145,147,148,149,150,151,152,153,154,155,159,160,161,162,163,164,165,167,168,170,171,172,173,174,175,176,177,178,179,180,181,182,183,187,188,189,190,192,195,196,197,198,199,201,204,209,211,212,213,214,217,218,219,220,221,222,223,224,226,228^ (17 different questionnaires), and 30 studies^35,36,37,40,42,43,44,47,48,49,50,53,54,55,57,130,138,141,143,146,169,184,185,186,191,193,203,205,227,229^ used a structured or semistructured diagnostic interview (8 types). One study used both.^27^ Studies using screening tools had a higher pooled prevalence than those using diagnostic interviews (24.4% [95% CI, 21.2%-27.8%; 153 studies^28,29,30,31,32,33,34,38,39,41,45,46,51,52,59,60,61,62,63,64,65,66,67,68,69,70,71,72,73,74,75,76,77,78,79,80,81,82,83,84,85,86,87,88,89,90,91,92,93,94,95,96,97,99,100,101,102,103,104,105,106,107,108,109,110,111,112,113,116,117,118,119,120,121,122,123,124,125,126,127,128,129,131,132,133,134,135,136,137,139,140,142,144,145,147,148,149,150,151,152,153,154,155,159,160,161,162,163,164,165,167,168,170,171,172,173,174,175,176,177,178,179,180,181,182,183,188,189,190,192,195,196,197,198,199,201,204,209,211,212,213,214,217,218,219,220,221,222,223,224,226,228^; n = 159 248] vs 11.5% [95% CI, 8.6%-14.9%; 30 studies^35,36,37,40,42,43,44,47,48,49,50,53,54,55,57,130,138,141,143,146,169,184,185,186,191,193,203,205,227,229^; n = 14 084]; *P* < .001) (eTable 3 in Supplement 1). By setting, prevalence was highest among studies performed in teaching hospitals (32.2%; 95% CI, 24.2%-40.7%; 42 studies^27,41,60,67,68,77,84,110,112,119,121,128,133,135,136,143,144,145,159,162,163,165,174,176,179,181,183,186,188,189,190,193,195,196,198,204,213,214,220,222,224,226^; n = 21 222) and lowest among studies from district or regional hospitals (14.4%; 95% CI, 11.1%-18.0%; 50 studies^28,34,36,42,51,57,61,62,63,64,65,70,72,73,74,76,79,81,91,93,95,100,101,104,108,111,113,116,122,123,125,130,131,132,139,140,146,153,155,172,173,178,185,187,191,197,201,209,218,227^; n = 47 166) (eTable 3 in Supplement 1).

The window of our search strategy included the COVID-19 pandemic. Thus, we were able to pool estimates of perinatal anxiety from studies that specifically examined the impact of the pandemic on perinatal anxiety prevalence. We found 60 studies^39,61,64,66,71,74,75,76,77,78,79,80,81,82,83,84,87,89,90,92,93,95,96,97,99,101,104,105,117,119,123,124,125,128,131,132,134,135,136,137,140,147,148,149,151,152,160,162,170,171,174,181,188,209,217,218,220,221,222,224,228^ from 18 countries that investigated the effect of the COVID-19 pandemic on perinatal generalized anxiety disorder. We found, however, that estimates were similar to the general population (prevalence, 24.3% [95% CI, 18.4%-30.7%; n = 63 334] vs 21.1% [95% CI, 18.2%-24.2%; 126 studies; n = 110 219]; *P* = .36) (eTable 3 in Supplement 1).

Using an adapted version of the Newcastle-Ottawa Scale, most studies were found to be of moderate methodological quality. There was considerable variation in study design, sampled populations, instruments used, and cutoff values of screening tools. To investigate this variation further, we excluded studies assessed as having a high risk of bias (scoring <5 out of 7). Excluding 81 studies, the pooled prevalence of generalized anxiety across the remaining 103 studies was 18.8% (95% CI, 16.1%-21.7%; n = 100 893; *P* = .01) (eTable 4 in Supplement 1).

## Discussion

We performed a systematic review and meta-analysis to assess the prevalence of 6 anxiety and related disorders among perinatal women in LMICs. We included 203 studies, and to our knowledge, this meta-analysis is the largest to examine perinatal anxiety disorders among women living in LMICs.

We found that anxiety and related disorders are common during pregnancy and in the 12 months following birth. Generalized anxiety disorder was the most prevalent anxiety disorder, with an estimated prevalence of 1 in 5 perinatal women. Posttraumatic stress disorder had an estimated prevalence of approximately 1 in 12 perinatal women. Adjustment disorder was least prevalent, found in 3 in 100 perinatal women. Perinatal depression has an estimated prevalence of 25% among women living in LMICs.^230^ Our findings highlight that anxiety disorders may be almost as common as perinatal depression in LMICs, yet they receive substantially less attention.^6^ Furthermore, our findings show that anxiety disorders may add significantly to the burden of perinatal mental health disorders in LMICs, and this burden needs urgent attention. The integration of perinatal mental health care into universal maternal and child health services is crucial.^231,232^ Screening and treatment options are needed that are culturally appropriate and that cover the breadth of mental health disorders, including anxiety and related disorders, and not simply depression. Evidence-based public health interventions could lead to a substantially reduced burden of perinatal mental health disorders.^13^

Our findings are broadly in keeping with previous reviews. A meta-analysis by Nielsen-Scott et al^11^ found the prevalence of anxiety symptoms in LMICs to be 29.2% antenatally and 24.4% postnatally. The data search for their review was conducted in 2020, identified 54 studies, and examined anxiety alone. In our review, the data search was updated through September 7, 2023; includes 203 studies; and, for the first time, examines a raft of anxiety and related disorders among women in LMICs. A 2017 review found a prevalence of perinatal anxiety in high-income countries of 19.4% antenatally and 13.7% postnatally,^6^ similar to a meta-analysis by Fawcett et al^12^ that examined the prevalence of combined perinatal anxiety disorders (including generalized anxiety disorder, obsessive-compulsive disorder, panic disorder, social anxiety disorder, agoraphobia, specific phobia, and posttraumatic stress disorder) globally and found an overall prevalence of 20.7%. However, this review included 26 studies, with only 4 from LMICs. In contrast, our review exclusively assessed LMICs and included a larger number of studies that examined anxiety and related disorders. Hence, it provides a more comprehensive picture of the burden that the many different anxiety and related disorders place on perinatal women in LMICs.

Rates of posttraumatic stress disorder are thought to be higher in LMICs than in high-income countries.^3^ Studies conducted in high-income countries have estimated prevalence to be 2%.^233^ We found a pooled prevalence of 8.3%, which is slightly higher than previous global estimates of 4% to 8%.^3,234^ We included studies that examined populations with experiences of intimate partner violence, conflict, and adolescent pregnancy. There were insufficient data to stratify by these factors; however, it is possible that in populations with greater exposure to such determinants, rates of perinatal posttraumatic stress disorder might be higher.

We found the pooled prevalence of obsessive-compulsive disorder to be 6.9%, which is higher than reported in previous meta-analyses. A 2013 meta-analysis found the prevalence of perinatal obsessive-compulsive disorder to be 2.1% antenatally and 2.4% postnatally.^2^ It included 17 studies, 6 of which were conducted in LMICs. The authors concluded that women were at higher risk of experiencing obsessive-compulsive disorder in the perinatal period than other times in their lives. Other studies have also suggested that the perinatal period may precipitate or exacerbate obsessive-compulsive disorder; however, evidence is limited, and results have been mixed.^3,235^

Numerous studies specifically examined the impact of the COVID-19 pandemic on perinatal women. We found that the prevalence of generalized anxiety disorder, however, was no different compared with studies not investigating the pandemic. This finding was surprising, as the World Health Organization found that the COVID-19 pandemic worsened mental health outcomes for women both during pregnancy and postnatally.^236^

Most studies included in our review used self-reported screening tools to assess anxiety disorders. Previous meta-analyses have found rates of perinatal anxiety to be higher when assessed with self-reported screening tools compared with diagnostic interviews.^11,237^ A 2017 review found the prevalence of antenatal anxiety to be 22.9% when assessed with a screening tool compared with 4.1% when assessed with diagnostic interviews.^6^ Our subgroup analysis showed a similar trend, with generalized anxiety prevalence estimated at 24.4% for screening tools and 11.5% for diagnostic interviews. In LMICs, however, diagnostic interviews are often not feasible due to a lack of resources and health care workers with adequate mental health training.^17,238^ In this case, validated screening tools can provide a method of systematic case identification and allow for monitoring of progress where other options may not be available.^6,13^ Furthermore, as these data suggest, screening tools allow far larger numbers of the population to be sampled and may therefore provide more representative results.

As perinatal anxiety disorders have important ramifications for a woman’s mental and physical health, as well as that of her child, recognizing them as a public health priority is crucial. The United Nations Sustainable Development Goals highlight the need to promote mental health and reduce maternal mortality.^239^ Therefore, action is required to embed mental health care into routine perinatal care, to include perinatal mental health in policy, and to develop culturally appropriate interventions and services.^17^

### Strengths and Limitations

The main strength of our study is the large number of studies we included, offering a comprehensive survey of perinatal anxiety disorders in LMICs around the globe. We included a number of anxiety disorders rather than generalized anxiety disorder alone. We investigated less common perinatal mental health disorders, including obsessive-compulsive disorder, posttraumatic stress disorder, social anxiety disorder, panic disorder, and adjustment disorder. In doing so, we have provided an extensive review of the prevalence of perinatal anxiety and related disorders in LMICs. Furthermore, we performed a subgroup analysis of generalized anxiety disorder, reporting its prevalence by country income status, regions, method of assessment, and setting.

Our study also has some limitations. An important limitation is that we only included studies published in English. Furthermore, we used a broad search strategy, aiming to capture a range of perinatal mental health disorders, which may have resulted in missed studies that focused specifically on rare disorders. Our study is also limited by variation in study design and methodological rigor among included studies. Many studies were cross-sectional and, hence, had a lower level of evidence than studies with prospectively collected data. Thus, we performed sensitivity analyses that excluded studies deemed at high risk of bias. The findings showed a reduced pooled point prevalence estimate of generalized anxiety disorder by almost 8 percentage points, highlighting the association of risk of bias with outcome assessment. Furthermore, the variation in prevalence estimates of generalized anxiety disorder found with diagnostic interviews compared with screening tools is an important consideration. The majority of the studies included in this meta-analysis used screening tools to quantify prevalence; therefore, these results should be interpreted with caution given the propensity of screening tools to overestimate prevalence.^5,6^

## Conclusions

The majority of perinatal women worldwide live in LMICs. This systematic review and meta-analysis highlights the substantial burden that anxiety and related disorders may place on these women. Perinatal mental health is essential for improving outcomes for women and their children and for reducing the inequities between high-income countries and LMICs.

## Notes

Supplement 1. **eMethods 1.** Search Strategy for MEDLINE Ovid **eMethods 2.** World Bank Country Income Classification List 2020 **eMethods 3.** Newcastle-Ottawa Scale (Modified Version) **eTable 1.** Risk-of-Bias Assessment **eTable 2.** Table of All Included Studies **eTable 3.** Generalized Anxiety Disorder Subgroup Analysis **eTable 4.** Subgroup Analysis of Risk of Bias
eReferences
**eFigure 1.** Forest Plot Anxiety Disorder **eFigure 2.** Forest Plot Posttraumatic Stress Disorder **eFigure 3.** Forest Plot Obsessive-Compulsive Disorder **eFigure 4.** Forest Plot Panic Disorder **eFigure 5.** Forest Plot Social Anxiety Disorder **eFigure 6.** Forest Plot Adjustment Disorder **eFigure 7.** Map–Number of Studies by Country

Supplement 2.
Data Sharing Statement
