# Supplementary material for: Prevalence of Perinatal Anxiety and Related Disorders in Low- and Middle-Income Countries: A Systematic Review and Meta-Analysis
Source: JAMA Netw Open. 2023 Nov 17;6(11):e2343711. doi: 10.1001/jamanetworkopen.2023.43711 (PMC10656650; doi:10.1001/jamanetworkopen.2023.43711)
Supplement: Supplement 1. — eMethods 1. Search Strategy for MEDLINE Ovid eMethods 2. World Bank Country Income Classification List 2020 eMethods 3. Newcastle-Ottawa Scale (Modified Version) eTable 1. Risk-of-Bias Assessment eTable 2. Table of All Included Studies eTable 3. Generalized Anxiety Disorder Subgroup Analysis eTable 4. Subgroup Analysis of Risk of Bias eReferences eFigure 1. Forest Plot Anxiety Disorder eFigure 2. Forest Plot Posttraumatic Stress Disorder eFigure 3. Forest Plot Obsessive-Compulsive Disorder eFigure 4. Forest Plot Panic Disorder eFigure 5. Forest Plot Social Anxiety Disorder eFigure 6. Forest Plot Adjustment Disorder eFigure 7. Map–Number of Studies by Country [file jamanetwopen-e2343711-s001.pdf]

Supplemental Online Content

Roddy Mitchell A, Gordon H, Atkinson J, et al. Prevalence of perinatal anxiety and related disorders in low- and middle-income countries: a systematic review and meta-analysis. *JAMA Netw Open*. 2023;6(11):e2343711. doi:10.1001/jamanetworkopen.2023.43711

eMethods 1. Search Strategy for MEDLINE Ovid

eMethods 2. World Bank Country Income Classification List 2020

eMethods 3. Newcastle-Ottawa Scale (Modified Version)

eTable 1. Risk-of-Bias Assessment

eTable 2. Table of All Included Studies

eTable 3. Generalized Anxiety Disorder Subgroup Analysis

eTable 4. Subgroup Analysis of Risk of Bias

eReferences

eFigure 1. Forest Plot Anxiety Disorder

eFigure 2. Forest Plot Posttraumatic Stress Disorder

eFigure 3. Forest Plot Obsessive-Compulsive Disorder

eFigure 4. Forest Plot Panic Disorder

eFigure 5. Forest Plot Social Anxiety Disorder

eFigure 6. Forest Plot Adjustment Disorder

eFigure 7. Map–Number of Studies by Country

This supplemental material has been provided by the authors to give readers additional information about their work.

© 2023 Roddy Mitchell A et al. *JAMA Network Open.*

eMethods 1. Search Strategy for MEDLINE Ovid

Order	Search terms
1	Perinatal.mp.
2	(Postnat* OR postpartum).mp.
3	(Pregnan* OR antenat*).mp.
4	1 OR 2 OR 3

Order	Search terms
5	(Mental illness*).mp.
6	(Mental health).mp.
7	(Mental disorder*).mp
8	Psychiatr*.mp.
9	Depression.mp.
10	Anxiety.mp.
11	Bipolar.mp.
12	Schizophreni*.mp.
13	Psycho?i*.mp.
14	5 OR 6 OR 7 OR 8 OR 9 OR 10 OR 11 OR 12 OR 13

Order	Search terms
15	Factor*.mp
16	Determinant*.mp
17	Prevalence.mp
18	Predictor*.mp
19	Rate*.mp
20	Risk*.mp
21	Incidence*.mp
22	15 OR 16 OR 17 OR 18 OR 19 OR 20 OR 21

Order	Search terms
23	EPOC LMIC filter (2020 v.4)
24	4 AND 14 AND 22 AND 23
25	Limit 24 to English language

eMethods 1. Search Strategy for MEDLINE Ovid (continued)

EPOC LMIC filters 2020 (v.4)

Based on the World Bank list of economies 2019:

Filters are based on the World Bank list of countries (2019), classified as low-income, lower-middle-income or upper-middle-income economies:
https://datahelpdesk.worldbank.org/knowledgebase/articles/906519-world-bank-country-and-lending-groups

MEDLINE (Ovid)

(**afghanistan** OR **albania** OR **algeria** OR **american samoa** OR **angola** OR **"antigua and barbuda"** OR antigua OR barbuda OR **argentina** OR **armenia** OR armenian OR **aruba** OR **azerbaijan** OR **bahrain** OR **bangladesh** OR **barbados** OR **republic of belarus** OR belarus OR byelarus OR belorussia OR byelorussian OR **belize** OR british honduras OR **benin** OR dahomey OR **bhutan** OR **bolivia** OR **"bosnia and herzegovina"** OR bosnia OR herzegovina OR **botswana** OR bechuanaland OR **brazil** OR brasil OR **bulgaria** OR **burkina faso** OR burkina fasso OR upper volta OR **burundi** OR urundi OR **cabo verde** OR cape verde OR **cambodia** OR kampuchea OR khmer republic OR **cameroon** OR cameron OR cameroun OR **central african republic** OR ubangi shari OR **chad** OR **chile** OR **china** OR **colombia** OR **comoros** OR comoro islands OR iles comores OR mayotte OR **democratic republic of the congo** OR democratic republic congo OR **congo** OR zaire OR **costa rica** OR **"cote d'ivoire"** OR "cote d' ivoire" OR cote divoire OR cote d ivoire OR ivory coast OR **croatia** OR **cuba** OR **cyprus** OR **czech republic** OR czechoslovakia OR **djibouti** OR french somaliland OR **dominica** OR **dominican republic** OR **ecuador** OR **egypt** OR united arab republic OR **el salvador** OR **equatorial guinea** OR spanish guinea OR **eritrea** OR **estonia** OR eswatini OR **swaziland** OR **ethiopia** OR **fiji** OR **gabon** OR gabonese republic OR **gambia** OR **"georgia (republic)"** OR georgian OR **ghana** OR gold coast OR **gibraltar** OR **greece** OR **grenada** OR **guam** OR **guatemala** OR **guinea** OR **guinea bissau** OR **guyana** OR british guiana OR **haiti** OR hispaniola OR **honduras** OR **hungary** OR **india** OR **indonesia** OR timor OR **iran** OR **iraq** OR isle of man OR **jamaica** OR **jordan** OR **kazakhstan** OR kazakh OR **kenya** OR "**democratic people's republic of korea"** OR **republic of korea** OR north korea OR south korea OR korea OR **kosovo** OR **kyrgyzstan** OR kirghizia OR kirgizstan OR kyrgyz republic OR kirghiz OR **laos** OR lao pdr OR "lao people's democratic republic" OR **latvia** OR **lebanon** OR lebanese republic OR **lesotho** OR basutoland OR **liberia** OR **libya** OR libyan arab jamahiriya OR **lithuania** OR **macau** OR macao OR **republic of north macedonia** OR macedonia OR **madagascar** OR malagasy republic OR **malawi** OR nyasaland OR **malaysia** OR malay federation OR malaya federation OR maldives OR **indian ocean islands** OR indian ocean OR **mali** OR **malta** OR **micronesia** OR federated states of micronesia OR kiribati OR marshall islands OR nauru OR northern mariana islands OR **palau** OR tuvalu OR **mauritania** OR **mauritius** OR **mexico** OR **moldova** OR moldovian OR **mongolia** OR **montenegro** OR **morocco** OR ifni OR **mozambique** OR portuguese east africa OR **myanmar** OR burma OR **namibia** OR **nepal** OR **netherlands antilles** OR **nicaragua** OR **niger** OR **nigeria** OR **oman** OR muscat OR **pakistan** OR **panama** OR **papua new guinea** OR new guinea OR **paraguay** OR **peru** OR **philippines** OR philipines OR phillipines OR phillippines OR **poland** OR "polish people's republic" OR **portugal** OR

© 2023 Roddy Mitchell A et al. *JAMA Network Open.*

eMethods 1. Search Strategy for MEDLINE Ovid (continued)

portuguese republic OR **puerto rico** OR **romania** OR **russia** OR russian federation OR ussr OR soviet union OR union of soviet socialist republics OR **rwanda** OR ruanda OR **samoa** OR pacific islands OR polynesia OR samoan islands OR navigator island OR navigator islands OR **"sao tome and principe"** OR **saudi arabia** OR **senegal** OR **serbia** OR **seychelles** OR **sierra leone** OR **slovakia** OR slovak republic OR **slovenia** OR **melanesia** OR solomon island OR solomon islands OR norfolk island OR norfolk islands OR **somalia** OR **south africa** OR **south sudan** OR **sri lanka** OR ceylon OR **"saint kitts and nevis"** OR "st. kitts and nevis" OR **saint lucia** OR "st. lucia" OR **"saint vincent and the grenadines"** OR saint vincent OR "st. vincent" OR grenadines OR **sudan** OR **suriname** OR surinam OR dutch guiana OR netherlands guiana OR **syria** OR syrian arab republic OR **tajikistan** OR tadjikistan OR tadzhikistan OR tadzhik OR **tanzania** OR tanganyika OR **thailand** OR siam OR **timor leste** OR east timor OR **togo** OR togolese republic OR **tonga** OR **"trinidad and tobago"** OR trinidad OR tobago OR **tunisia** OR **turkey** OR **turkmenistan** OR turkmen OR **uganda** OR **ukraine** OR **uruguay** OR **uzbekistan** OR uzbek OR **vanuatu** OR new hebrides OR **venezuela** OR **vietnam** OR viet nam OR **middle east** OR west bank OR gaza OR palestine OR **yemen** OR **yugoslavia** OR **zambia** OR **zimbabwe** OR northern rhodesia OR global south OR **africa south of the sahara** OR sub-saharan africa OR subsaharan africa OR **africa, central** OR central africa OR **africa, northern** OR north africa OR northern africa OR magreb OR maghrib OR sahara OR **africa, southern** OR southern africa OR **africa, eastern** OR east africa OR eastern africa OR **africa, western** OR west africa OR western africa OR **west indies** OR **indian ocean islands** OR **caribbean** OR **central america** OR **latin america** OR "south and central america" OR **south america** OR **asia, central** OR central asia OR **asia, northern** OR north asia OR northern asia OR **asia, southeastern** OR southeastern asia OR south eastern asia OR southeast asia OR south east asia OR **asia, western** OR western asia OR **europe, eastern** OR east europe OR eastern europe OR developing country OR **developing countries** OR developing nation? OR developing population? OR developing world OR less developed countr* OR less developed nation? OR less developed population? OR less developed world OR lesser developed countr* OR lesser developed nation? OR lesser developed population? OR lesser developed world OR under developed countr* OR under developed nation? OR under developed population? OR under developed world OR underdeveloped countr* OR underdeveloped nation? OR underdeveloped population? OR underdeveloped world OR middle income countr* OR middle income nation? OR middle income population? OR low income countr* OR low income nation? OR low income population? OR lower income countr* OR lower income nation? OR lower income population? OR underserved countr* OR underserved nation? OR underserved population? OR underserved world OR under served countr* OR under served nation? OR under served population? OR under served world OR deprived countr* OR deprived nation? OR deprived population? OR deprived world OR poor countr* OR poor nation? OR poor population? OR poor world OR poorer countr* OR poorer nation? OR poorer population? OR poorer world OR developing econom* OR less developed econom* OR lesser developed econom* OR under developed econom* OR underdeveloped econom* OR middle income econom* OR low income econom* OR lower income econom* OR low gdp OR low gnp OR low gross domestic OR low gross national OR lower gdp OR lower gnp OR lower gross domestic OR lower gross national OR lmic OR lmics OR third world OR lami countr* OR transitional countr* OR emerging economies OR emerging nation?)**.ti,ab,sh,kf.**

© 2023 Roddy Mitchell A et al. *JAMA Network Open.*

eMethods 2. World Bank Country Income Classification List 2020

Low-Income Countries

Afghanistan
Burkina Faso
Burundi
Central African Republic
Chad
Congo, Democratic Republic
Eritrea
Ethiopia
Gambia, The
Guinea
Guinea-Bissau
Haiti
Korea, Democratic People's Republic
Liberia
Madagascar
Malawi
Mali
Mozambique
Niger
Rwanda
Sierra Leone
Somalia
South Sudan
Sudan
Syrian Arab Republic
Tajikistan
Togo
Uganda
Yemen, Republic

Lower-Middle Income Countries

Angola
Algeria
Bangladesh
Benin
Bhutan
Bolivia
Cabo Verde
Cambodia
Cameroon
Comoros
Congo, Republic
Côte d'Ivoire
Djibouti
Egypt, Arab Republic
El Salvador
Eswatini
Ghana
Honduras
India
Kenya
Kiribati
Kyrgyz Republic
Lao PDR
Lesotho
Mauritania
Micronesia, Federated States
Moldova
Mongolia
Morocco
Myanmar
Nepal
Nicaragua
Nigeria
Pakistan
Papua New Guinea
Philippines
São Tomé and Principe
Senegal
Solomon Islands
Sri Lanka
Tanzania
Timor-Leste
Tunisia
Ukraine
Uzbekistan
Vanuatu
Vietnam
West Bank and Gaza
Zambia
Zimbabwe

Upper-middle income countries

Albania
American Samoa
Argentina
Armenia
Azerbaijan
Belarus
Belize
Bosnia and Herzegovina
Botswana
Brazil
Bulgaria
China
Colombia
Costa Rica
Cuba
Dominica

© 2023 Roddy Mitchell A et al. *JAMA Network Open.*

eMethods 2. World Bank Country Income Classification List 2020 (continued)

Dominican Republic
Equatorial Guinea
Ecuador
Fiji
Gabon
Georgia
Grenada
Guatemala
Guyana
Indonesia
Iran, Islamic Republic
Iraq
Jamaica
Jordan
Kazakhstan
Kosovo
Lebanon
Libya
Malaysia
Maldives
Marshall Islands
Mexico
Montenegro
Namibia
North Macedonia
Paraguay
Peru
Russian Federation
Samoa
Serbia
South Africa
St. Lucia
St. Vincent and the Grenadines
Suriname
Thailand
Tonga
Turkey
Turkmenistan
Tuvalu
Venezuela, Republic Bolivarian

© 2023 Roddy Mitchell A et al. *JAMA Network Open.*

eMethods 3. Newcastle-Ottawa Scale (Modified Version)

Selection

- Representativeness of the exposed cohort
  1. Truly/somewhat representative of the average perinatal woman in the community
  0. Selected group of perinatal women/no description of the derivation of the cohort

- Selection of the non-exposed cohort
  1. Drawn from the same community as the expose cohort
  0. Drawn from a different source/no description of the derivation of the non-exposed cohort

- Ascertainment of the exposure
  1. Secure record
  0. Self-reported/no description

Comparability

- Comparability of the cohorts on the basis of the design or analysis
  1. Study controls for important factors so that exposed and non-exposed are comparable
  0. Exposed and non-exposed differ by important factor/no description of comparability

Outcome

- Assessment of outcome
  1. Diagnostic interview
  0. Self-reported/no description

- Was follow-up long enough for outcomes to occur
  1. Yes (antenatal ≥ 6 weeks' gestation, postnatal ≥ 2 weeks postpartum)
  0. No

- Adequacy of follow-up of cohort
  1. Complete follow-up of participants/≥80% follow-up of participants
  0. <80% follow-up of participants/no statement

TOTAL score out of 7

© 2023 Roddy Mitchell A et al. *JAMA Network Open.*

eTable 1. Risk-of-Bias Assessment

Domains:
1: Representativeness of the exposed cohort
2: Selection of the non-exposed cohort
3: Ascertainment of the exposure
4: Comparability of the cohorts on the basis of the design or analysis
5: Assessment of outcome
6: Was follow-up long enough for outcomes to occur
7: Adequacy of follow-up of cohort

Author	D1	D2	D3	D4	D5	D6	D7	Overall
Aba	Low	Low	Low	Low	High	High	Low	Low
Abdelhai	High	Low	Low	Low	High	Low	Low	Low
Abiodun	Low	Low	Low	High	Low	High	Low	Low
Abrahams	Low	Low	Low	High	Low	High	Low	Low
Ade-Ojo	High	Low	Low	Low	High	High	Low	High
Aderibigbe	High	High	Low	High	High	Low	Low	High
Adewuya	Low	Low	Low	Low	Low	Low	Low	Low
Agbaje	High	Low	Low	Low	High	Low	Low	Low
Ahmed a	Low	Low	Low	Low	High	Low	Low	Low
Ahmed b	High	Low	Low	Low	High	High	Low	High
Ali	Low	Low	Low	High	High	High	Low	High
Alipour	High	Low	Low	High	High	High	Low	High
Aryal	High	Low	Low	Low	High	Low	Low	Low
Ayaz	High	Low	Low	High	High	Low	High	High
Azlan	High	Low	Low	Low	High	High	Low	High
Bachani	High	Low	Low	Low	High	Low	High	High
Bante	Low	Low	Low	Low	High	Low	Low	Low
Basha	Low	Low	Low	Low	High	High	Low	Low
Bataineh	Low	Low	Low	Low	High	High	Low	Low
Begum	Low	Low	Low	Low	High	Low	High	Low
Beketie	Low	Low	Low	Low	High	High	Low	Low
Bhushan	Low	Low	Low	Low	High	High	Low	Low
Bindt a	Low	Low	Low	Low	High	Low	Low	Low
Bindt b	Low	Low	Low	Low	High	Low	Low	Low
Bishaw	Low	Low	Low	High	High	High	Low	High
Boggaram	Low	Low	Low	Low	Low	High	Low	Low
CaglayanKeles	High	Low	High	High	High	High	Low	High
Cankaya	Low	Low	Low	High	High	High	Low	High
Cao	High	Low	High	Low	High	High	Low	High
Castroe Couto	Low	Low	Low	High	Low	Low	High	Low
Chen a	High	Low	Low	High	High	Low	Low	High
Chen b	High	Low	Low	Low	High	High	Low	High
Choi	High	Low	Low	Low	High	Low	Low	Low
Coelho	Low	Low	Low	Low	Low	Low	Low	Low
Costa	Low	Low	Low	Low	High	Low	Low	Low
Cui	High	Low	Low	Low	High	High	Low	High
da Silva	Low	Low	Low	Low	High	High	Low	Low
de Matos	Low	Low	Low	Low	Low	High	Low	Low
de Mello	High	Low	Low	Low	High	High	Low	High
Dias	Low	Low	Low	Low	Low	Low	Low	Low
Dikmen-Yildiz a	Low	Low	Low	Low	High	Low	High	Low
Dikmen-Yildiz b	Low	Low	Low	Low	High	Low	Low	Low
Ding	Low	Low	High	Low	High	Low	Low	Low
Djatche Miafo	Low	Low	Low	Low	Low	High	Low	Low
Dong	High	Low	High	Low	High	High	Low	High

© 2023 Roddy Mitchell A et al. *JAMA Network Open.*

Author	D1	D2	D3	D4	D5	D6	D7	Overall
Duan	Low	Low	Low	Low	High	High	Low	Low
Edhborg	High	Low	Low	Low	High	Low	Low	Low
Effati-Daryani	High	High	Low	High	High	Low	High	High
Eleje	Low	Low	Low	Low	High	High	Low	Low
EsquivelLauzurique	Low	Low	Low	Low	High	Low	Low	Low
Fadzil	Low	Low	Low	Low	High	High	Low	Low
Faisal-Cury	High	Low	Low	Low	High	Low	Low	Low
Farias a	Low	Low	Low	Low	Low	High	Low	Low
Farias b	High	High	Low	High	Low	High	Low	High
Ferraro	Low	Low	Low	Low	Low	Low	High	Low
Fisher	Low	Low	Low	Low	Low	Low	Low	Low
Gankanda	Low	Low	Low	Low	High	Low	Low	Low
Gao	Low	Low	Low	Low	High	Low	Low	Low
Ge	High	Low	High	High	High	High	Low	High
Gelaye	Low	Low	Low	Low	High	Low	Low	Low
Gerges	High	High	High	Low	High	High	Low	High
Goyal	High	Low	Low	Low	Low	High	Low	Low
Guillen-Burgos	High	Low	High	Low	High	High	Low	High
Gul	Low	Low	Low	Low	High	Low	Low	Low
Guo	Low	Low	Low	Low	High	High	Low	Low
Hababa	High	Low	Low	Low	High	Low	Low	Low
Hou	High	Low	Low	Low	High	Low	High	High
Irum	High	Low	Low	High	High	High	Low	High
Jelly	High	Low	Low	High	High	Low	High	High
Jha	Low	Low	Low	Low	Low	Low	Low	Low
Jiang a	High	Low	High	Low	High	High	High	High
Jiang b	Low	Low	High	Low	High	Low	Low	Low
Jigeer	High	Low	Low	Low	High	High	Low	High
Jusoh	High	Low	Low	Low	High	Low	Low	Low
Kalok	High	Low	Low	High	High	High	Low	High
Kang	Low	Low	Low	Low	High	Low	Low	Low
Kantipudi	High	Low	Low	Low	High	High	Low	High
Kassaw	High	Low	Low	High	High	High	Low	High
Keramat	Low	Low	Low	Low	High	High	Low	Low
Keskin	Low	Low	Low	Low	High	High	Low	Low
Khan	High	Low	High	Low	Low	High	Low	High
Khatri	High	Low	Low	Low	High	Low	Low	Low
Koen	Low	Low	Low	Low	High	Low	High	Low
Koyucu	High	Low	High	High	High	High	Low	High
Kugbey	Low	Low	Low	Low	High	High	Low	Low
Lalani	Low	Low	Low	Low	High	Low	Low	Low
Lelisho	High	Low	Low	Low	High	High	Low	High
Lin	High	Low	High	Low	High	Low	Low	High
Liu a	Low	Low	Low	Low	High	Low	High	Low
Liu b	High	High	High	Low	High	High	Low	High
Liu c	High	High	High	High	High	High	Low	High
Lopes	Low	Low	Low	Low	High	High	Low	Low
Loret de Mola	Low	Low	Low	Low	High	High	Low	Low
Lu	Low	Low	Low	Low	High	High	Low	Low
Luo	High	Low	High	Low	High	High	Low	High
Luong	Low	Low	Low	Low	High	High	Low	Low
Ma a	Low	Low	Low	Low	High	Low	High	Low
Ma b	Low	Low	Low	Low	High	High	Low	Low
Ma c	High	Low	Low	Low	High	Low	Low	Low
Maharlouei	High	Low	High	High	High	High	High	High
Mahenge a	High	Low	Low	Low	High	High	Low	High
Mahenge b	Low	Low	Low	Low	High	High	Low	Low

© 2023 Roddy Mitchell A et al. *JAMA Network Open.*

Author	D1	D2	D3	D4	D5	D6	D7	Overall
Mahmoodi a	High	Low	Low	Low	High	Low	Low	Low
Mahmoodi b	High	Low	Low	Low	High	Low	High	High
Malaju	High	Low	Low	Low	High	Low	Low	Low
Maleki	High	Low	Low	Low	High	High	Low	High
Malemela	High	Low	Low	Low	High	High	Low	High
Mare	Low	Low	Low	Low	Low	Low	High	Low
Margawati	High	Low	High	Low	High	High	Low	High
Maria	Low	Low	Low	High	High	High	Low	High
Mateus	High	Low	High	High	High	High	Low	High
Mo	High	Low	High	High	High	High	Low	High
Mônica Mariade Jesus	High	Low	Low	Low	High	Low	Low	Low
Muller	Low	High	High	High	High	High	Low	High
Nampijja	Low	Low	Low	Low	Low	Low	Low	Low
Naseer	High	Low	Low	Low	Low	High	Low	Low
Nasreen a	Low	Low	Low	Low	High	Low	Low	Low
Nasreen b	Low	Low	Low	Low	High	Low	Low	Low
Nasreen c	Low	Low	Low	Low	High	Low	Low	Low
Nasreen d	High	Low	Low	Low	High	Low	High	High
Nazir	High	Low	Low	Low	High	High	Low	High
Ngocho	Low	Low	Low	Low	High	Low	Low	Low
Niaz	High	Low	Low	Low	High	High	Low	High
Niazi	High	Low	Low	Low	High	High	Low	High
Nomura	High	Low	Low	Low	High	High	Low	High
Nwafor	Low	Low	Low	Low	High	High	Low	Low
Odinka	High	Low	Low	Low	High	Low	Low	Low
Pabon	Low	Low	Low	Low	High	Low	Low	Low
Padilla	Low	Low	Low	Low	High	Low	High	Low
Pages	Low	Low	Low	Low	High	High	Low	Low
Paskulin	Low	Low	Low	Low	High	Low	Low	Low
Patabendige a	High	Low	Low	Low	High	High	Low	High
Patabendige b	Low	Low	Low	Low	High	Low	Low	Low
Peng	High	Low	Low	Low	High	Low	Low	Low
Peter	High	Low	Low	High	Low	High	High	High
Pinheiro	Low	Low	Low	Low	Low	High	Low	Low
Pise	High	Low	Low	Low	High	High	Low	High
Priyadarshanie	High	Low	Low	High	High	Low	Low	High
Qasrawi	High	Low	High	Low	High	High	Low	High
Qiao	High	Low	Low	Low	High	Low	Low	Low
Qin	High	Low	Low	Low	High	High	Low	High
Qu a	High	Low	Low	Low	High	High	Low	High
Qu b	High	Low	Low	Low	High	Low	Low	Low
Ray	High	Low	High	High	High	High	Low	High
Redinger a	Low	Low	Low	Low	High	Low	High	Low
Redinger b	High	Low	Low	Low	High	High	Low	High
Rees	Low	Low	Low	Low	High	Low	Low	Low
Ryali	Low	Low	Low	High	Low	High	Low	Low
Scholl	Low	Low	Low	Low	Low	High	Low	Low
Shaban	Low	Low	Low	Low	High	Low	Low	Low
Shagufta	High	High	High	High	High	High	Low	High
Shehroz	High	High	Low	High	High	High	Low	High
Shi	Low	Low	High	Low	High	Low	High	High
Shrestha a	High	Low	Low	Low	High	Low	Low	Low
Shrestha b	High	Low	Low	Low	High	High	Low	High
Silove	Low	Low	Low	Low	High	Low	Low	Low
Silva	Low	Low	Low	Low	Low	Low	Low	Low
Singh	Low	Low	Low	Low	High	High	Low	Low

© 2023 Roddy Mitchell A et al. *JAMA Network Open.*

Author	D1	D2	D3	D4	D5	D6	D7	Overall
Soares	Low	Low	Low	Low	High	Low	High	Low
Sut	High	Low	High	High	High	Low	Low	High
Takahasi	Low	Low	Low	Low	High	Low	Low	Low
Tang	High	Low	Low	Low	High	High	Low	High
Tariq	High	Low	Low	Low	Low	High	Low	Low
Tavares	Low	Low	Low	Low	Low	Low	Low	Low
Thurkkada	High	Low	High	Low	High	High	Low	High
Tibebu	Low	Low	Low	Low	High	High	Low	Low
Tikka	Low	Low	Low	Low	High	High	Low	Low
Ture	Low	Low	Low	Low	Low	Low	High	Low
Umuziga	Low	Low	Low	Low	High	High	Low	Low
van Heyningen a	High	Low	Low	Low	Low	High	Low	Low
van Heyningen b	Low	Low	Low	Low	Low	Low	Low	Low
Verbeek	High	Low	Low	Low	High	Low	Low	Low
Wang a	High	Low	High	High	High	Low	Low	High
Wang b	High	Low	High	Low	High	High	Low	High
Waqas	High	Low	Low	Low	High	High	Low	High
Wassif	High	Low	Low	Low	High	Low	Low	Low
Wegbom	Low	Low	Low	Low	High	High	Low	Low
Wu	Low	Low	Low	Low	High	High	Low	Low
Xie	Low	Low	High	Low	High	Low	Low	Low
Xu	High	Low	High	Low	High	High	Low	High
Yang a	Low	Low	High	Low	High	Low	Low	Low
Yang b	High	Low	High	Low	High	High	Low	High
Yang c	High	Low	Low	Low	High	High	Low	High
Yang d	Low	Low	Low	Low	High	High	Low	Low
Yang e	Low	Low	Low	Low	High	High	Low	Low
Yassa	High	Low	Low	Low	High	Low	Low	Low
Youseflu	High	Low	Low	Low	High	High	Low	High
Yu a	Low	Low	Low	Low	High	Low	High	Low
Yu b	High	Low	Low	Low	High	Low	High	High
Yue	High	Low	Low	Low	High	Low	Low	Low
Zainiyah	High	Low	High	Low	High	Low	High	High
Zambaldi a	Low	Low	Low	Low	Low	Low	Low	Low
Zambaldi b	Low	Low	Low	Low	Low	Low	Low	Low
Zeng a	High	Low	Low	Low	High	High	Low	High
Zeng b	High	Low	Low	Low	High	High	High	High
Zhang a	High	Low	High	Low	High	Low	Low	High
Zhang b	High	Low	Low	Low	High	Low	High	High
Zhang c	Low	Low	High	High	High	High	Low	High
Zhang d	High	Low	High	Low	High	High	Low	High
Zhang e	High	Low	High	Low	High	High	Low	High
Zhou a	High	High	High	High	High	High	Low	High
Zhou b	High	Low	Low	Low	High	Low	Low	Low
Zhou c	Low	Low	Low	Low	High	High	Low	Low

© 2023 Roddy Mitchell A et al. *JAMA Network Open.*

eTable 1. Risk-of-Bias Assessment (continued)

Duplicate surname list of references

Author	reference	Author	reference	Author	reference	Author	reference
Ahmed a	161	Ma a	81	Shrestha a	149	Zeng a	78
Ahmed b	92	Ma b	64	Shrestha b	147	Zeng b	85
Bindt a	145	Ma c	46	van Heyningen a	178	Zhang a	71
Bindt b	146	Mahmoodi a	128	van Heyningen b	180	Zhang b	83
Chen a	34	Mahmoodi b	129	Wang a	67	Zhang c	51
Chen b	44	Nasreen a	4	Wang b	48	Zhang d	72
Dikmen-Yildiz a	199	Nasreen b	5	Yang a	70	Zhang e	42
Dikmen-Yildiz b	200	Nasreen c	137	Yang b	39	Zhou a	79
Farias a	23	Nasreen d	134	Yang c	41	Zhou b	52
Farias b	10	Patabendige a	184	Yang d	50	Zhou c	43
Jiang a	60	Patabendige b	182	Yang e	40		
Jiang b	59	Qu a	87	Yu a	76		
Liu a	73	Qu b	88	Yu b	84		
Liu b	74	Redinger a	174	Zambaldi a	29		
Liu c	62	Redinger b	177	Zambaldi b	31		

© 2023 Roddy Mitchell A et al. *JAMA Network Open.*

eTable 2. Table of All Included Studies

Author	Year	Title	Study design	Tool used	N population	NOS RoB score
**Afghanistan**						
Niazi[1]	**2023**	Prevalence and associated factors of depression, anxiety, and stress among pregnant women in Herat, Afghanistan: A cross-sectional study	Cross-sectional study	Depression Anxiety Stress Scale	691	4
**Bangladesh**						
Begum[2]	2021	Prevalence and Associated Factors of Antenatal Anxiety Symptoms in Bangladesh: A Repeated Measures Cluster Data Analysis	Prospective cohort study	The State-Trait Anxiety Inventory	1360	5
Edhborg[3]	2011	Impact of postpartum depressive and anxiety symptoms on mothers' emotional tie to their infants 2-3 months postpartum: A population-based study from rural Bangladesh	Prospective cohort study	The State-Trait Anxiety Inventory	671	5
Nasreen[4]	2011	Prevalence and associated factors of depressive and anxiety symptoms during pregnancy: A population based study in rural Bangladesh	Cross-sectional study	The State-Trait Anxiety Inventory	720	6
Nasreen[5]	2010	Low birth weight in offspring of women with depressive and anxiety symptoms during pregnancy: results from a population based study in Bangladesh	Prospective cohort study	The State-Trait Anxiety Inventory	583	6
**Brazil**						
Lopes[6]	2023	Perceived stress and associated factors in pregnant women: a cross-sectional study nested within a population-based cohort	Prospective cohort study	The State-Trait Anxiety Inventory	1279	5
Loret de Mola[7]	2023	Sleep and its association with depressive and anxiety symptoms during the last weeks of pregnancy: A population-based study	Cross-sectional study	Generalised Anxiety Disorder-7	2307	5
Scholl[8]	2022	Are adolescents more likely to have antenatal anxiety disorders than adult women? A comparison between two samples	Cross-sectional study	Mini International Neuropsychiatric Interview	1852	6

© 2023 Roddy Mitchell A et al. *JAMA Network Open.*

Author	Year	Title	Study design	Tool used	N population	NOS RoB score
de Mello[9]	2021	Generalized Anxiety Disorder, Depressive Symptoms and the Occurrence of Stressors Events in a Probabilistic Sample of Pregnant Women	Cross-sectional study	Generalized Anxiety Disorder-7	980	4
Farias[10]	2021	Maternal mental health and gestational weight gain in a Brazilian Cohort	Prospective cohort study	Mini International Neuropsychiatric Interview	188	3
Muller[11]	2021	Prevalence of anxiety and depression disorder and associated factors during postpartum in puerperal women	Cross-sectional study	Beck Anxiety Scale	250	2
Nomura[12]	2021	Impact of the covid-19 pandemic on maternal anxiety in Brazil	Cross-sectional study	Beck Anxiety Scale	1662	4
Soares[13]	2021	Suicide risk and prematurity: A study with pregnant adolescents	Prospective cohort study	Mini International Neuropsychiatric Interview	645	5
Pabon[14]	2020	Overall Maternal Morbidity during Pregnancy Identified with the WHO-WOICE Instrument	Cross-sectional study	Generalized Anxiety Disorder-7	531	6
Costa[15]	2018	Mental disorders in pregnancy and newborn conditions: longitudinal study with pregnant women attended in primary care	Prospective cohort study	Obsessive Compulsive Inventory	300	6
de Matos[16]	2018	The perception of parental bonding in pregnant adolescents and its association with generalized anxiety disorder	Cross-sectional study	Mini International Neuropsychiatric Interview	870	6
Ferraro[17]	2017	The specific and combined role of domestic violence and mental health disorders during pregnancy on new-born health	Prospective cohort study	Mini International Neuropsychiatric Interview	775	6
Mônica Mariade Jesus[18]	2017	Anxiety in pregnancy: prevalence and associated factors	Cross-sectional study	Hospital Anxiety and Depression Scale	209	5
Paskulin[19]	2017	Association between dietary patterns and mental disorders in pregnant women in Southern Brazil	Cross-sectional study	Patient Health Questionnaire	712	6
Peter[20]	2017	Association between perceived social support and anxiety in pregnant adolescents	Randomised control trial	Mini International Neuropsychiatric Interview	871	3
Castroe Couto[21]	2016	Antenatal depression: Prevalence and risk factor patterns across the gestational period	Prospective cohort study	Mini International Neuropsychiatric Interview	148	5

© 2023 Roddy Mitchell A et al. *JAMA Network Open.*

Author	Year	Title	Study design	Tool used	N population	NOS RoB score
Coelho[22]	2014	Parental bonding and suicidality in pregnant teenagers: a population-based study in southern Brazil	Cross-sectional study	Mini International Neuropsychiatric Interview	828	7
Farias[23]	2013	Prevalence of psychiatric disorders in the first trimester of pregnancy and factors associated with current suicide risk	Cross-sectional study	Mini International Neuropsychiatric Interview	239	6
Takahasi[24]	2013	Mental health and physical inactivity during pregnancy: a cross-sectional study nested in the BRISA cohort study	Cross-sectional study	Beck Anxiety Scale	1447	6
da Silva[25]	2012	Suicidality and associated factors in pregnant women in Brazil	Cross-sectional study	Hospital Anxiety and Depression Scale	1334	5
Pinheiro[26]	2012	Suicidal behavior in pregnant teenagers in southern Brazil: Social, obstetric and psychiatric correlates	Cross-sectional study	Mini International Neuropsychiatric Interview	828	6
Tavares[27]	2012	Prevalence of suicide risk and comorbidities in postpartum women in Pelotas	Cross-sectional study	Mini International Neuropsychiatric Interview	919	7
Dias[28]	2011	Pregnancy is associated with psychiatric symptoms in a low-income countryside community of Brazil	Cross-sectional study	Mini International Neuropsychiatric Interview	94	7
Zambaldi[29]	2011	Bio-socio-demographic factors associated with post-traumatic stress disorder in a sample of postpartum Brazilian women	Cross-sectional study	Mini International Neuropsychiatric Interview	400	7
Silva[30]	2010	Relationship between religious practice, alcohol use, and psychiatric disorders among pregnant women. [Portuguese, English]	Cross-sectional study	Mini International Neuropsychiatric Interview	260	7
Zambaldi[31]	2009	Postpartum obsessive-compulsive disorder: prevalence and clinical characteristics	Cross-sectional study	Mini International Neuropsychiatric Interview	400	7
Faisal-Cury[32]	2007	Prevalence of anxiety and depression during pregnancy in a private setting sample	Cross-sectional study	The State-Trait Anxiety Inventory	432	5
**Cameroon**						
Djatche Miafo[33]	2023	Epidemiological profile of perinatal mental disorders at a tertiary hospital in Yaounde-Cameroon	Cross-sectional study	The State-Trait Anxiety Inventory	194	6

© 2023 Roddy Mitchell A et al. *JAMA Network Open.*

Author	Year	Title	Study design	Tool used	N population	NOS RoB score
**China**						
Chen[34]	2023	Anxiety and depression among perinatal women during the long-term normal prevention of COVID-19 pandemic period in China: a cross-sectional study	Cross-sectional study	Self Rating Anxiety Scale	1338	4
Gao[35]	2023	Latent Trajectories and Risk Factors of Prenatal Stress, Anxiety, and Depression in Southwestern China-A Longitudinal Study	Prospective cohort study	Hamilton Anxiety Scale	916	6
Hou[36]	2023	Joint effects of recent stressful life events and adverse childhood experiences on perinatal comorbid anxiety and depression	Prospective cohort study	Generalised Anxiety Disorder-7	757	4
Qin[37]	2023	Prevalence and risk factors of anxious and depressive symptoms in first-trimester females and their partners: a study during the pandemic era of COVID-19 in China	Cross-sectional study	Generalised Anxiety Disorder-7	169	4
Shi[38]	2023	Maternal affective and stress-related factors during pregnancy affect the occurrence of childhood allergic diseases: A Shanghai MCPC study	Prospective cohort study	Self Rating Anxiety Scale	2979	4
Yang[39]	2023	Prevalence of and relevant factors for depression and anxiety symptoms among pregnant women on the eastern seaboard of China in the post-COVID-19 era: a cross sectional study	Cross-sectional study	Generalised Anxiety Disorder-7	1963	3
Yang[40]	2023	Anxiety symptoms and health-related quality of life in mainland Chinese pregnant women: a cross-sectional study	Cross-sectional study	Self Rating Anxiety Scale	770	5
Yang[41]	2023	Mediating effect of mindfulness level on the relationship between marital quality and postpartum depression among primiparas	Cross-sectional study	Self Rating Anxiety Scale	121	4
Zhang[42]	2023	The association between paternal childcare involvement and postpartum depression and anxiety among Chinese women-a path model analysis	Cross-sectional study	Generalised Anxiety Disorder-7	778	3

© 2023 Roddy Mitchell A et al. *JAMA Network Open.*

Author	Year	Title	Study design	Tool used	N population	NOS RoB score
Zhou[43]	2023	Effects of maternal pre-pregnancy body mass index and gestational weight gain on antenatal mental disorders in China: a prospective study	Prospective cohort study	Self Rating Anxiety Scale	4890	5
Chen[44]	2022	COVID-19 affects psychological symptoms of pregnant women indirectly by increasing their maternal concerns	Cross-sectional study	Generalised Anxiety Disorder-7	828	4
Jigeer[45]	2022	Association of residential noise exposure with maternal anxiety and depression in late pregnancy	Cross-sectional study	Self Rating Anxiety Scale	2018	4
Ma[46]	2022	Association of maternal prenatal depression and anxiety with toddler sleep: the China-Anhui Birth Cohort study	Prospective cohort study	Self Rating Anxiety Scale	1583	5
Pages[47]	2022	The Impact of COVID-19 on Maternal Mental Health during Pregnancy: A Comparison between Canada and China within the CONCEPTION Cohort	Cross-sectional study	Generalised Anxiety Disorder-7	484	5
Wang[48]	2022	Pregnant Women's Anxiety and Depression Symptoms and Influence Factors in the COVID-19 Pandemic in Changzhou, China	Cross-sectional study	Generalised Anxiety Disorder-7	681	3
Wu[49]	2022	Association between intimate partner violence and prenatal anxiety and depression in pregnant women: a cross-sectional survey during the COVID-19 epidemic in Shenzhen, China	Cross-sectional study	Generalised Anxiety Disorder-7	3434	5
Yang[50]	2022	Incidence and correlates of insomnia and its impact on health-related quality of life among Chinese pregnant women: a cross-sectional study	Cross-sectional study	Self Rating Anxiety Scale	717	5
Zhang[51]	2022	The impact of COVID-19 on the mental health of pregnant women in Shanghai, China	Cross-sectional study	Generalised Anxiety Disorder-7	365	3
Zhou[52]	2022	The prevalence and associated factors of prenatal depression and anxiety in twin pregnancy: a cross-sectional study in Chongqing, China	Cross-sectional study	Self Rating Anxiety Scale	210	5

© 2023 Roddy Mitchell A et al. *JAMA Network Open.*

Author	Year	Title	Study design	Tool used	N population	NOS RoB score
Cao[53]	2021	Pregnant women's psychological state and influence factors: anxiety, and depression during COVID-19 outbreak	Cross-sectional study	Self Rating Anxiety Scale	256	3
Cui[54]	2021	Prenatal anxiety and the associated factors among Chinese pregnant women during the COVID-19 pandemic——a smartphone questionnaire survey study	Cross-sectional study	Generalised Anxiety Disorder-7	304	4
Ding[55]	2021	Knowledge, attitudes, practices, and influencing factors of anxiety among pregnant women in Wuhan during the outbreak of COVID-19: a cross-sectional study	Cross-sectional study	Self Rating Anxiety Scale	817	5
Dong[56]	2021	Investigation on the mental health status of pregnant women in China during the Pandemic of COVID-19	Cross-sectional study	Self Rating Anxiety Scale	156	3
Ge[57]	2021	Anxiety and Adaptation of Behavior in Pregnant Zhuang Women During the COVID-19 Pandemic: A Mixed-Mode Survey	Cross-sectional study	Self Rating Anxiety Scale	446	2
Guo[58]	2021	The prevalence of and factors associated with antenatal depression among all pregnant women first attending antenatal care: a cross-sectional study in a comprehensive teaching hospital	Cross-sectional study	Generalized Anxiety Disorder-7	5728	5
Jiang[59]	2021	Perinatal mental health problems in rural China: The role of social factors	Cross-sectional study	Depression Anxiety Stress Scale	1027	5
Jiang[60]	2021	The mental health status and approaches of accessing antenatal care information among pregnant women during COVID-19 epidemic：a cross-sectional study in China	Cross-sectional study	Self Rating Anxiety Scale	1873	2
Lin[61]	2021	Sleep Conditions Associate with Anxiety and Depression Symptoms among Pregnant Women during the Epidemic of COVID-19 in Shenzhen	Cross-sectional study	Self Rating Anxiety Scale	751	4
Liu[62]	2021	The Prevalence of Psychological Symptoms in Pregnant Healthcare Workers (HCWs) and Pregnant Non-HCWs During the Early Stage of COVID-19 Pandemic in Chongqing, China	Cross-sectional study	John Hopkins Symptoms Checklist	410	1

© 2023 Roddy Mitchell A et al. *JAMA Network Open.*

Author	Year	Title	Study design	Tool used	N population	NOS RoB score
Luo[63]	2021	Comorbid Anxiety and Depression and Related Factors Among Pregnant and Postpartum Chinese Women During the Coronavirus Disease 2019 Pandemic	Cross-sectional study	Generalized Anxiety Disorder-7	97	3
Ma[64]	2021	Resilience mediates the effect of self-efficacy on symptoms of prenatal anxiety among pregnant women: a nationwide smartphone cross-sectional study in China	Cross-sectional study	Generalized Anxiety Disorder-7	665	5
Mo[65]	2021	Association of Perceived Threat, Negative Emotions, and Self-Efficacy With Mental Health and Personal Protective Behavior Among Chinese Pregnant Women During the COVID-19 Pandemic: Cross-sectional Survey Study	Cross-sectional study	Generalized Anxiety Disorder-7	4087	2
Peng[66]	2021	A multi-center survey on the postpartum mental health of mothers and attachment to their neonates during COVID-19 in Hubei Province of China	Cross-sectional study	Self Rating Anxiety Scale	71	5
Wang[67]	2021	Mental health and preventive behaviour of pregnant women in China during the early phase of the COVID-19 period	Cross-sectional study	Generalized Anxiety Disorder-7	15428	3
Xie[68]	2021	Alteration in the psychologic status and family environment of pregnant women before and during the COVID-19 pandemic	Cross-sectional study	Symptom Checklist Generalized Anxiety Disorder-70 (revised)	2657	5
Xu[69]	2021	Mental health among pregnant women under public health interventions during COVID-19 outbreak in Wuhan, China	Cross-sectional study	Self Rating Anxiety Scale	274	3
Yang[70]	2021	Social, Cognitive, and eHealth Mechanisms of COVID-19-Related Lockdown and Mandatory Quarantine That Potentially Affect the Mental Health of Pregnant Women in China: Cross-Sectional Survey Study	Cross-sectional study	Generalized Anxiety Disorder-7	19515	5
Zhang[71]	2021	Psychobehavioral Responses, Post-Traumatic Stress and Depression in Pregnancy During the Early Phase of COVID-19 Outbreak	Cross-sectional study	Post Traumatic Stress Disorder Checklist	1901	4

© 2023 Roddy Mitchell A et al. *JAMA Network Open.*

Author	Year	Title	Study design	Tool used	N population	NOS RoB score
Zhang[72]	2021	Association of Sleep Duration and Screen Time With Anxiety of Pregnant Women During the COVID-19 Pandemic	Cross-sectional study	Self Rating Anxiety Scale	1794	3
Liu[73]	2020	Prevalence and Associated Factors of Postpartum Anxiety and Depression Symptoms Among Women in Shanghai, China	Cross-sectional study	Self Rating Anxiety Scale	1204	5
Liu[74]	2020	Prenatal anxiety and obstetric decisions among pregnant women in Wuhan and Chongqing during the COVID-19 outbreak: a cross-sectional study	Cross-sectional study	Self Rating Anxiety Scale	1947	2
Lu[75]	2020	Mental health outcomes among Chinese prenatal and postpartum women after the implementation of universal two-child policy	Cross-sectional study	Generalized Anxiety Disorder-7	3110	5
Yu[76]	2020	Prevalence of depression symptoms and its influencing factors among pregnant women in late pregnancy in urban areas of Hengyang City, Hunan Province, China: A cross-sectional study	Cross-sectional study	Generalized Anxiety Disorder-7	813	5
Yue[77]	2020	Association between social support and anxiety among pregnant women in the third trimester during the coronavirus disease 2019 (COVID-19) epidemic in Qingdao, China: The mediating effect of risk perception	Cross-sectional study	Self Rating Anxiety Scale	308	5
Zeng[78]	2020	Mental Health Outcomes in Perinatal Women During the Remission Phase of COVID-19 in China	Cross-sectional study	Generalized Anxiety Disorder-7	625	4
Zhou[79]	2020	The prevalence of psychiatric symptoms of pregnant and non-pregnant women during the COVID-19 epidemic	Cross-sectional study	Post Traumatic Stress Disorder Checklist & Generalized Anxiety Disorder-7	544	1
Duan[80]	2019	Relationship between trait neuroticism and suicidal ideation among postpartum women in China: Testing a mediation model	Cross-sectional study	Generalized Anxiety Disorder-7	1027	5
Ma[81]	2019	The impact of resilience on prenatal anxiety and depression among pregnant women in Shanghai	Prospective cohort study	Self Rating Anxiety Scale	2813	5

© 2023 Roddy Mitchell A et al. *JAMA Network Open.*

Author	Year	Title	Study design	Tool used	N population	NOS RoB score
Tang[82]	2019	Influencing factors for prenatal Stress, anxiety and depression in early pregnancy among women in Chongqing, China	Cross-sectional study	Hamilton Anxiety Scale	1220	4
Zhang[83]	2018	Prevalence and relevant factors of anxiety and depression among pregnant women in a cohort study from south-east China	Prospective cohort study	Self Rating Anxiety Scale	2150	4
Yu[84]	2017	Sleep was associated with depression and anxiety status during pregnancy: a prospective longitudinal study	Prospective cohort study	Self Rating Anxiety Scale	2115	4
Zeng[85]	2017	Retinoids, anxiety and peripartum depressive symptoms among Chinese women: a prospective cohort study	Prospective cohort study	Self Rating Anxiety Scale	156	3
Kang[86]	2016	Prevalence and Risk Factors of Maternal Anxiety in Late Pregnancy in China	Cross-sectional study	Self Rating Anxiety Scale	467	6
Qu[87]	2012	Posttraumatic stress disorder and depression among new mothers at 8 months later of the 2008 Sichuan earthquake in China	Cross-sectional study	Impact of Event Scale	317	4
Qu[88]	2012	The impact of the catastrophic earthquake in China's Sichuan province on the mental health of pregnant women	Cross-sectional study	Impact of Event Scale	311	5
Qiao[89]	2009	The prevalence and related risk factors of anxiety and depression symptoms among Chinese pregnant women in Shanghai	Cross-sectional study	Hospital Anxiety and Depression Scale	527	5
**Colombia**						
Guillen-Burgos[90]	2023	Pregnancy and mental health outcomes during the COVID-19 pandemic in Colombia: A nationwide cross-sectional study	Cross-sectional study	Generalised Anxiety Disorder-7 & Post Traumatic Stress Disorder Checklist	721	3
**Cuba**						
Esquivel Lauzurique[91]	2022	Prevalence, Incidence, and Persistence of Postpartum Anxiety, Depression, and Comorbidity: A Cohort Study Among Women in Havana Cuba	Prospective cohort study	The State-Trait Anxiety Inventory	281	6

© 2023 Roddy Mitchell A et al. *JAMA Network Open.*

Author	Year	Title	Study design	Tool used	N population	NOS RoB score
**Egypt**						
Ahmed[92]	2022	Correlation between psychiatric impact of COVID-19 during pregnancy and fetal outcomes in Egyptian women	Cross-sectional study	The State-Trait Anxiety Inventory & Post Traumatic Stress Disorder Checklist	238	5
Wassif[93]	2019	Assessment of Postpartum Depression and Anxiety among Females Attending Primary Health Care Facilities in Qaliubeya Governorate, Egypt	Cross-sectional study	Depression Anxiety Stress Scale	500	5
Abdelhai[94]	2015	Screening for antepartum anxiety and depression and their association with domestic violence among Egyptian pregnant women	Cross-sectional study	Hospital Anxiety and Depression Scale	376	5
**Ethiopia**						
Malaju[95]	2023	A structural equation modelling of the direct and indirect factors associated with functional status over time as measured by WHODAS-32 items among postpartum women in Northwest Ethiopia	Prospective cohort study	Depression Anxiety Stress Scale & Post Traumatic Stress Disorder Checklist	775	5
Tibebu[96]	2023	Depression, anxiety and stress among HIV-positive pregnant women in Ethiopia during the COVID-19 pandemic	Cross-sectional study	Depression Anxiety Stress Scale	423	5
Bishaw[97]	2022	Generalized Anxiety Disorder and Its Associated Factors Among Pregnant Women During COVID-19 at Public Health Facilities of East Gojjam Zone, 2020: A Multi-Center Cross-Sectional Study	Cross-sectional study	Generalized Anxiety Disorder-7	806	4
Lelisho[98]	2022	Generalized anxiety disorder among mothers attending perinatal services during COVID-19 pandemic: using ordinal logistic regression model	Cross-sectional study	Generalized Anxiety Disorder-7	423	4
Bante[99]	2021	Comorbid anxiety and depression: Prevalence and associated factors among pregnant women in Arba Minch zuria district, Gamo zone, southern Ethiopia	Cross-sectional study	Generalized Anxiety Disorder-7	667	6

© 2023 Roddy Mitchell A et al. *JAMA Network Open.*

Author	Year	Title	Study design	Tool used	N population	NOS RoB score
Beketie[100]	2021	Magnitude and associated factors of antenatal depression among mothers attending antenatal care in Arba Minch town, Ethiopia, 2018	Cross-sectional study	Generalized Anxiety Disorder-7	316	5
Kassaw[101]	2020	The prevalence of general anxiety disorder and its associated factors among women's attending at the perinatal service of Dilla University referral hospital, Dilla town, Ethiopia, April, 2020 in Covid pandemic	Cross-sectional study	Generalized Anxiety Disorder-7	178	3
**Ghana**						
Kugbey[102]	2021	Prevalence and Correlates of Prenatal Depression, Anxiety and Suicidal Behaviours in the Volta Region of Ghana	Cross-sectional study	Hospital Anxiety and Depression Scale	214	5
**India**						
Ryali[103]	2023	Is cesarean section a clinical marker for psychiatric and sleep disorder in young mothers? A cross-sectional study from rural South India	Cross-sectional study	Mini International Neuropsychiatric Interview	245	5
Bachani[104]	2022	Anxiety and depression among women with COVID-19 infection during childbirth-experience from a tertiary care academic center	Prospective cohort study	Generalised Anxiety Disorder-7	243	4
Nazir[105]	2022	Emotional difficulties in pregnant females who tested positive for COVID-19: A cross-sectional study from South Kashmir, India	Cross-sectional study	Depression Anxiety Stress Scale	63	4
Tiwari[106]	2022	Generalized Anxiety Disorder and Factors Affecting It during the Postnatal Period: An Observational Study	Prospective cohort study	Generalised Anxiety Disorder-7	180	4
Ray[107]	2022	A Cross-sectional Study to Assess the Anxiety and Depression among Perinatal Mothers during the COVID-19 Pandemic	Cross-sectional study	Generalised Anxiety Disorder-7	124	2
Singh[108]	2022	Knowledge and Anxiety of Pregnant Women towards COVID-19 Pandemic in the Prevaccination Phase	Cross-sectional study	Generalised Anxiety Disorder-7	280	5

© 2023 Roddy Mitchell A et al. *JAMA Network Open.*

Author	Year	Title	Study design	Tool used	N population	NOS RoB score
Thurkkada[109]	2022	Prenatal Anxiety, Perceived Stress, and Coping Behaviour regarding Covid-19 among Pregnant Women at a selected Hospital, Kochi, South India	Cross-sectional study	Beck Anxiety Scale	384	3
Jelly[110]	2021	Impact of COVID-19 Pandemic on the Psychological Status of Pregnant Women	Cross-sectional study	Generalized Anxiety Disorder-7	333	3
Jha[111]	2021	Prevalence of Common Mental Disorders among pregnant women-Evidence from population-based study in rural Haryana, India	Prospective cohort study	Generalised Anxiety Disorder-7	457	7
Maria[112]	2021	Prevalence and Determinants of Postpartum Anxiety among Women Availing Health Services at a Rural Maternity Hospital in South India	Cross-sectional study	Generalised Anxiety Disorder-7	231	4
Tikka[113]	2021	Anxiety among pregnant women during the COVID-19 pandemic in India - A multicentric study	Cross-sectional study	Generalised Anxiety Disorder-7	620	5
Ture[114]	2021	Magnitude and determinants of psychological morbidities among pregnant women: Results from a pregnancy cohort in rural Central India	Cross-sectional study	Global Mental Health Assessment Tool - PrimaryCare Version	650	6
Bhushan[115]	2020	The association between social support through contacts with Accredited Social Health Activists (ASHAs) and antenatal anxiety among women in Mysore, India: a cross-sectional study	Cross-sectional study	Edinburgh Postnatal Depression subscale 3A	480	5
Goyal[116]	2020	Psychiatric Morbidity, Cultural Factors, and Health-Seeking Behaviour in Perinatal Women: A Cross-Sectional Study from a Tertiary Care Centre of North India	Cross-sectional study	Mini International Neuropsychiatric Interview	281	5
Kantipudi[117]	2020	Antenatal Depression and Generalized Anxiety Disorder in a Tertiary Hospital in South India	Cross-sectional study	Generalized Anxiety Disorder-7	209	4
Khatri[118]	2020	Psychological status of pregnant women during COVID-19 pandemic: A cross-sectional study from Mumbai	Cross-sectional study	Generalized Anxiety Disorder-7	66	5
Boggaram[119]	2017	An exploratory study of identification of psychiatric disorders during pregnancy	Cross-sectional study	Mini International Neuropsychiatric Interview	100	6

© 2023 Roddy Mitchell A et al. *JAMA Network Open.*

Author	Year	Title	Study design	Tool used	N population	NOS RoB score
**Indonesia**						
Margawati[120]	2022	Anxiety among Pregnant Women in Rural-Urban Area Indonesia during the COVID-19 Pandemic in Semarang, Indonesia	Cross-sectional study	Hamilton Anxiety Scale	238	3
Zainiyah[121]	2020	Anxiety in Pregnant Women During Coronavirus (Covid-19) Pandemic in East Java, Indonesia	Cross-sectional study	Depression Anxiety Stress Scale	70	3
**Iran**						
Maleki[122]	2022	Influential factors of general anxiety disorder among Iranian pregnant women during the second peak of COVID-19 pandemic	Cross-sectional study	Generalised Anxiety Disorder-7	2336	4
Keramat[123]	2021	Factors influencing stress, anxiety, and depression among Iranian pregnant women: the role of sexual distress and genital self-image	Cross-sectional study	Depression Anxiety Stress Scale	295	5
Maharlouei[124]	2021	Depression and anxiety among pregnant mothers in the initial stage of the Coronavirus Disease (COVID-19) pandemic in the southwest of Iran	Cross-sectional study	Depression Anxiety Stress Scale	540	1
Effati-Daryani[125]	2020	Depression, stress, anxiety and their predictors in Iranian pregnant women during the outbreak of COVID-19	Cross-sectional study	Depression Anxiety Stress Scale	205	2
Youseflu[126]	2020	The role of reproductive variables, anxiety, physical activity, on the sleep quality of lactating women referring to health care centers of Zanjan-Iran	Cross-sectional study	Back Anxiety Inventory	380	4
Alipour[127]	2018	Psychological profiles of risk for antenatal depression and anxiety in Iranian sociocultural context	Cross-sectional study	General Health Questionnaire	296	3
Mahmoodi[128]	2017	Mother-father differences in postnatal psychological distress and its determinants in Iran	Cross-sectional study	General Health Questionnaire	124	5
Mahmoodi[129]	2016	Correlation between Kind of Delivery and Posttraumatic Stress Disorder	Prospective cohort study	Post Traumatic Stress Disorder Symptom Scale	240	4
Shaban[130]	2013	Post-Traumatic Stress Disorder (PTSD) Following Childbirth: Prevalence and Contributing Factors	Cross-sectional study	Post Traumatic Stress Disorder Symptom Scale	600	6

© 2023 Roddy Mitchell A et al. *JAMA Network Open.*

Author	Year	Title	Study design	Tool used	N population	NOS RoB score
**Jordan**						
Bataineh[131]	2022	Impact of coronavirus 2019 pandemic on post-traumatic stress disorder symptoms among pregnant women in Jordan	Cross-sectional study	Impact of Event Scale	481	5
Basha[132]	2021	Prevalence of anxiety among pregnant women attending antenatal care in Jordan: A single center study	Cross-sectional study	Generalised Anxiety Disorder-7	200	5
**Lebanon**						
Gerges[133]	2023	Traversing mental health disorders during pregnancy: Lebanese women's experiences of antepartum depression and anxiety	Cross-sectional study	Lebanese Anxiety Scale-10	433	2
**Malaysia**						
Nasreen[134]	2022	Impact of parental perinatal depressive and anxiety symptoms trajectories on early parent-infant impaired bonding: a cohort study in east and west coasts of Malaysia	Prospective cohort study	Depression Anxiety Stress Scale	566	4
Kolak[135]	2022	COVID-19 Pandemic and Maternal Psychological Wellbeing During the Malaysian Movement Control Order: A Cross-Sectional Study	Cross-sectional study	Depression Anxiety Stress Scale	415	3
Azlan[136]	2020	Anxiety, depression and marital satisfaction in women with hyperemesis gravidarum: a comparative cross-sectional study in Hospital Tengku Ampuan Rahimah, Klang, Malaysia	Cross-sectional study	Depression Anxiety Stress Scale	62	4
Nasreen[137]	2018	Prevalence and determinants of antepartum depressive and anxiety symptoms in expectant mothers and fathers: results from a perinatal psychiatric morbidity cohort study in the east and west coasts of Malaysia	Prospective cohort study	Depression Anxiety Stress Scale	904	6
Jusoh[138]	2014	Anxiety symptoms and associated factors among outpatient antenatal mother: A cross sectional study at University Malaya Medical Centre, Malaysia	Cross-sectional study	Hospital Anxiety and Depression Scale	320	5

© 2023 Roddy Mitchell A et al. *JAMA Network Open.*

Author	Year	Title	Study design	Tool used	N population	NOS RoB score
Fadzil[139]	2013	Risk factors for depression and anxiety among pregnant women in Hospital Tuanku Bainun, Ipoh, Malaysia	Cross-sectional study	Mini International Neuropsychiatric Interview	175	5
**Mexico**						
Padilla[140]	2021	Perinatal anxiety symptoms: Rates and risk factors in Mexican women	Prospective cohort study	Symptom Checklist Generalized Anxiety Disorder-70 (revised)	280	5
**Morocco**						
Hababa[141]	2023	Measurement of maternal morbidity during postpartum with the WHO-WOICE tools in Morocco	Cross-sectional study	Generalised Anxiety Disorder-7	253	5
**Mozambique**						
Khan[142]	2022	Women's mental health in Mozambique: is maternity a protective factor?	Cross-sectional study	Mini International Neuropsychiatric Interview	220	4
**Multinational**						
Mateus[143]	2022	Rates of depressive and anxiety symptoms in the perinatal period during the COVID-19 pandemic: Comparisons between countries and with pre-pandemic data	Cross-sectional study	Generalized Anxiety Disorder-7	1217	2
Qasrawi[144]	2022	Machine learning techniques for predicting depression and anxiety in pregnant and postpartum women during the COVID-19 pandemic: A cross-sectional regional study	Cross-sectional study	Generalized Anxiety Disorder-7	961	3
Bindt[145]	2013	No association between antenatal common mental disorders in low-obstetric risk women and adverse birth outcomes in their offspring: results from the CDS study in Ghana and Cote D'Ivoire	Prospective cohort study	Generalized Anxiety Disorder-7	717	6
Bindt[146]	2012	Antepartum Depression and Anxiety Associated with Disability in African Women: Cross-Sectional Results from the CDS Study in Ghana and Cote d'Ivoire	Cross-sectional study	Generalized Anxiety Disorder-7	1018	6

Author	Year	Title	Study design	Tool used	N population	NOS RoB score
**Nepal**						
Shrestha[147]	2021	Anxiety among pregnant women about corona virus infections during covid-19 pandemic at a tertiary care center in Nepal: A descriptive cross-sectional study	Cross-sectional study	Hamilton Anxiety Scale	273	4
Aryal[148]	2018	Anxiety and Depression among Pregnant Women and Mothers of Children Under one Year in Sindupalchowk District	Cross-sectional study	John Hopkins Symptom Checklist	567	5
Shrestha[149]	2014	Factors associated with post-natal anxiety among primiparous mothers in Nepal	Cross-sectional study	The State-Trait Anxiety Inventory	216	5
**Nicaragua**						
Verbeek[150]	2015	Anxiety and depression during pregnancy in Central America: A cross-sectional study among pregnant women in the developing country Nicaragua	Cross-sectional study	The State-Trait Anxiety Inventory	98	5
**Nigeria**						
Eleje[151]	2023	Depression, anxiety, and stress and adverse pregnancy outcomes in pregnant women with history of recurrent pregnancy loss in Nigeria	Case-control study	Depression Anxiety Stress Scale	94	5
Wegbom[152]	2023	Determinants of Depression, Anxiety, and Stress among Pregnant Women Attending Tertiary Hospitals in Urban Centers, Nigeria	Cross-sectional study	Depression Anxiety Stress Scale	413	5
Ade-Ojo[153]	2022	Comparison of Anxiety and Depression Among HIV-Positive and HIV-Negative Pregnant Women During COVID-19 Pandemic in Ekiti State, Southwest Nigeria	Cross-sectional study	Generalised Anxiety Disorder-7	198	4
Nwafor[154]	2021	Prevalence and predictors of depression, anxiety, and stress symptoms among pregnant women during COVID-19-related lockdown in Abakaliki, Nigeria	Cross-sectional study	Depression Anxiety Stress Scale	456	5
Agbaje[155]	2019	Depressive and anxiety symptoms and associated factors among postnatal women in Enugu-North Senatorial District, South-East Nigeria: a cross-sectional study	Cross-sectional study	Hospital Anxiety and Depression Scale	267	5

© 2023 Roddy Mitchell A et al. *JAMA Network Open.*

Author	Year	Title	Study design	Tool used	N population	NOS RoB score
Odinka[156]	2019	Socio-demographic correlates of postpartum psychological distress among apparently healthy mothers in two tertiary hospitals in Enugu, South-East Nigeria	Cross-sectional study	Hospital Anxiety and Depression Scale	309	5
Adewuya[157]	2006	Anxiety disorders among Nigerian women in late pregnancy: a controlled study	Cross-sectional study	Mini International Neuropsychiatric Interview	172	7
Abiodun[158]	1993	Psychiatric morbidity in a pregnant population in Nigeria	Prospective cohort study	Present State Examination Schedule	240	5
Aderibigbe[159]	1992	The validity of the 28-item General Health Questionnaire in a Nigerian antenatal clinic	Cross-sectional study	General Health Questionnaire	106	3
**Pakistan**						
Lalani[160]	2023	Individual and collective contribution of antenatal psychosocial distress conditions and preterm birth in Pakistani women	Prospective cohort study	The State-Trait Anxiety Inventory	1603	6
Ahmed[161]	2022	Antenatal anxiety and depression: Frequency and correlates during the COVID-19 pandemic in Pakistan	Cross-sectional study	Hospital Anxiety and Depression Scale	390	6
Irum[162]	2022	Frequency and Risk Factors of Anxiety and Depression among Pregnant Women in Abbottabad, Pakistan: A Facility-Based Cross-Sectional Study	Cross-sectional study	Hospital Anxiety and Depression Scale	200	3
Gul[163]	2019	Antenatal anxiety and depression among pregnant women attending tertiary care hospital, Mardan, Pakistan	Cross-sectional study	Hamilton Anxiety Scale	212	6
Naseer[164]	2019	Occurrence of psychiatric Disorders Among Pregnant Females	Cross-sectional study	Present State Examination Schedule	200	6
Shagufta[165]	2019	Prevalence, Differences, and Predictors of Anxiety and Depression among Pregnant and Non-Pregnant Women in Peshawar Khyber Pakhtunkhwa Pakistan	Cross-sectional study	Hospital Anxiety and Depression Scale	150	1
Shehroz[166]	2019	Depression and anxiety during pregnancy period	Cross-sectional study	Interview with ICD-Depression Anxiety Stress Scale criteria	200	2

© 2023 Roddy Mitchell A et al. *JAMA Network Open.*

Author	Year	Title	Study design	Tool used	N population	NOS RoB score
Tariq[167]	2019	To determine the frequency of panic disorders and social phobia in teenage pregnancy presenting in a tertiary hospital in Pakistan	Cross-sectional study	Interview with ICD-Depression Anxiety Stress Scale criteria	150	5
Waqas[168]	2015	Psychosocial factors of antenatal anxiety and depression in Pakistan: is social support a mediator?	Cross-sectional study	Hospital Anxiety and Depression Scale	500	4
Ali[169]	2012	Frequency and associated factors for anxiety and depression in pregnant women: a hospital-based cross-sectional study	Cross-sectional study	Aga Khan Anxiety and Depression Scale	167	4
Niaz[170]	2004	Anxiety and depression in pregnant women presenting in the OPD of a teaching hospital	Cross-sectional study	Hospital Anxiety and Depression Scale	200	4
**Peru**						
Gelaye[171]	2020	Association of antepartum depression, generalized anxiety, and posttraumatic stress disorder with infant birth weight and gestational age at delivery	Prospective cohort study	Post Traumatic Stress Disorder Checklist & Generalized Anxiety Disorder-7	4408	6
**Rwanda**						
Umuziga[172]	2020	A cross-sectional study of the prevalence and factors associated with symptoms of perinatal depression and anxiety in Rwanda	Cross-sectional study	Self Rating Anxiety Scale	77	5
**South Africa**						
Mare[173]	2021	Perinatal suicidality: prevalence and correlates in a South African birth cohort	Prospective cohort study	Mini International Neuropsychiatric Interview	522	6
Redinger[174]	2020	Antenatal depression and anxiety across pregnancy in urban South Africa	Prospective cohort study	The State-Trait Anxiety Inventory	704	5
Malemela[175]	2019	The Prevalence of Obsessive-Compulsive Disorder Symptoms and their Psychological Correlates amongst Pregnant Clinic Attendees in the Capricorn District, South Africa	Cross-sectional study	Obsessive Compulsive Inventory	206	4
Abrahams[176]	2018	Factors associated with household food insecurity and depression in pregnant South African women from a low socio-economic setting: a cross-sectional study	Cross-sectional study	Mini International Neuropsychiatric Interview	376	5

© 2023 Roddy Mitchell A et al. *JAMA Network Open.*

Author	Year	Title	Study design	Tool used	N population	NOS RoB score
Redinger[177]	2018	First trimester antenatal depression and anxiety: prevalence and associated factors in an urban population in Soweto, South Africa	Cross-sectional study	The State-Trait Anxiety Inventory	945	4
van Heyningen[178]	2018	Comparison of mental health screening tools for detecting antenatal depression and anxiety disorders in South African women	Cross-sectional study	Mini International Neuropsychiatric Interview	376	5
Koen[179]	2017	Maternal posttraumatic stress disorder and infant developmental outcomes in a South African birth cohort study	Prospective cohort study	Mini International Neuropsychiatric Interview	111	5
van Heyningen[180]	2017	Prevalence and predictors of anxiety disorders amongst low-income pregnant women in urban South Africa: a cross-sectional study	Cross-sectional study	Mini International Neuropsychiatric Interview	376	7
Choi[181]	2015	Maladaptive coping mediates the influence of childhood trauma on depression and PTSD among pregnant women in South Africa	Cross-sectional study	Davidson Trauma Scale	84	5
**Sri Lanka**						
Patabendige[182]	2022	The sustained adverse impact of COVID-19 pandemic on mental health among pregnant women in Sri Lanka: a reassessment during the second wave	Cross-sectional study	Hospital Anxiety and Depression Scale	311	6
Gankanda[183]	2021	Prevalence and associated factors of post-traumatic stress disorder (PTSD) among a cohort of Sri Lankan post-partum mothers: a cross-sectional study	Prospective cohort study	PTSD Symptom Scale-Self Report (PSS-SR)	225	6
Patabendige[184]	2020	Psychological impact of the COVID-19 pandemic among pregnant women in Sri Lanka	Cross-sectional study	Hospital Anxiety and Depression Scale	257	4
Priyadarshanie[185]	2020	Sinhala translation of the Perinatal Anxiety Screening Scale: A valid and reliable tool to detect anxiety disorders among antenatal women	Cross-sectional study	Perinatal Anxiety Screening Scale	221	4
**Tanzania**						
Ngocho[186]	2019	Depression and anxiety among pregnant women living with HIV in Kilimanjaro region, Tanzania	Cross-sectional study	Brief Symptom Index	199	6

© 2023 Roddy Mitchell A et al. *JAMA Network Open.*

Author	Year	Title	Study design	Tool used	N population	NOS RoB score
Mahenge[187]	2015	The prevalence of mental health morbidity and its associated factors among women attending a prenatal clinic in Tanzania	Cross-sectional study	Post Traumatic Diagnostic Scale &<br>John Hopkins Symptom Checklist	1180	4
Mahenge[188]	2013	Intimate partner violence during pregnancy and associated mental health symptoms among pregnant women in Tanzania: a cross-sectional study	Cross-sectional study	Post Traumatic Diagnostic Scale	1180	5
**Timor-Leste**						
Rees[189]	2016	A high-risk group of pregnant women with elevated levels of conflict-related trauma, intimate partner violence, symptoms of depression and other forms of mental distress in post-conflict Timor-Leste	Cross-sectional study	Harvard Trauma Questionnaire	1672	6
Silove[190]	2015	Pathways to perinatal depressive symptoms after mass conflict in Timor-Leste: a modelling analysis using cross-sectional data	Cross-sectional study	Harvard Trauma Questionnaire	387	6
**Türkiye**						
Caglayan Keles[191]	2023	The risk of anxiety and depression among pregnant women during the COVID-19 pandemic in Turkey: A cross-sectional online survey	Cross-sectional study	Hospital Anxiety and Depression Scale	164	2
Aba[192]	2022	Levels and Predictors of Anxiety and Depression in Turkish Pregnant Woman During the Covid-19 Pandemic	Cross-sectional study	Beck Anxiety Scale	269	5
Cankaya[193]	2022	Stress, anxiety, intolerance of uncertainty, and psychological well-being characteristics of pregnant women with and without threatened miscarriage: a case-control study	Case-control study	Depression Anxiety Stress Scale	201	4
Keskin[194]	2021	Mental disorders among pregnant women during the COVID-19 pandemic. A cross-sectional study	Cross-sectional study	Beck Anxiety Scale	356	5
Koyucu[195]	2021	The Covid 19 outbreak: Maternal Mental Health and Associated Factors	Cross-sectional study	Depression Anxiety Stress Scale	724	2

© 2023 Roddy Mitchell A et al. *JAMA Network Open.*

Author	Year	Title	Study design	Tool used	N population	NOS RoB score
Ayaz[196]	2020	Anxiety and depression symptoms in the same pregnant women before and during the COVID-19 pandemic	Prospective cohort study	Beck Anxiety Scale	63	3
Sut[197]	2020	Anxiety, depression, and related factors in pregnant women during the COVID-19 pandemic in Turkey: A web-based cross-sectional study	Cross-sectional study	Hospital Anxiety and Depression Scale	403	3
Yassa[198]	2020	Anxiety levels and obsessive compulsion symptoms of pregnant women during the COVID-19 pandemic	Cross-sectional study	The State-Trait Anxiety Inventory & Maudsley Obsessive-Compulsive Inventory	203	5
Dikmen-Yildiz [199]	2017	Factors associated with post-traumatic stress symptoms (PTSS) 4-6 weeks and 6 months after birth: A longitudinal population-based study	Prospective cohort study	Post Traumatic Diagnostic Scale	950	5
Dikmen-Yildiz [200]	2017	Depression, anxiety, PTSD and comorbidity in perinatal women in Turkey: A longitudinal population-based study	Prospective cohort study	Hospital Anxiety and Depression Scale	950	6
**Uganda**						
Nampijja[201]	2019	The burden and risk factors for postnatal depression and depressive symptomatology among women in Kampala	Cross-sectional study	Mini International Neuropsychiatric Interview	300	7
**Vietnam**						
Luong[202]	2021	Fear, anxiety and depression among pregnant women during COVID-19 pandemic: impacts of healthy eating behaviour and health literacy	Cross-sectional study	Generalised Anxiety Disorder-7	513	5
Fisher[203]	2010	Common perinatal mental disorders in northern Viet Nam: community prevalence and health care use	Cross-sectional study	Structured clinical interview with DSM-IV criteria	199	7

© 2023 Roddy Mitchell A et al. *JAMA Network Open.*

eTable 3. Generalized Anxiety Disorder Subgroup Analysis

	No. of studies	Total no. of participants	No. of participants with outcome	Prevalence % (95% CI)	p-value	I^2 (%)	tau^2
**Method of assessment**							
Self-reported screening tool	153	159 248	30 524	24.4 (21.2 – 27.8)	<0.001	99.6	0.24
Diagnostic interview	30	14 084	1 671	11.5 (8.6 – 14.9)		97.0	0.07
Combined	1	221	81	-		-	-
**Setting**							
Teaching hospital	42	21 222	7 119	32.2 (24.2 – 40.7)	<0.001	99.4	0.34
Secondary hospital	50	47 166	6 628	14.4 (11.1 – 18.0)		99.1	0.12
Community/primary health	71	58 763	11 928	21.1 (17.3 – 25.1)		99.3	0.19
Combination	21	46 402	6 601	28.4 (19.0 – 38.8)		99.8	0.24
**Study design**							
Cross sectional study	148	135 708	25 130	22.6 (19.4 – 26.0)	<0.001	99.5	0.24
Prospective cohort study	33	36 679	7 012	20.5 (15.1 – 26.6)		99.5	0.17
Randomised Control Trial (baseline data)	1	871	76	-		-	-
Case-control (control data)	2	295	58	20.5 (19.4 – 25.0)		65.8	0.02
**“At-risk” population**							
COVID-19	60	63 334	12 979	24.3 (18.4 – 30.7)	0.355	99.3	0.18
Not COVID-19 study	126	110 219	19 297	21.1 (18.2 – 24.2)		99.7	0.33

© 2023 Roddy Mitchell A et al. *JAMA Network Open.*

eTable 4. Subgroup Analysis by Risk of Bias

	No. of studies	Total no. of participants	No. of participants with outcome	Prevalence % (95% CI)	p-value	I^2 (%)	tau^2
**Generalised anxiety disorder**							
Low	103	100 893	16 018	18.8 (16.1 – 21.7)	0.010	99.2	0.14
High	81	72 660	16 258	26.6 (19.4 – 25.0)		99.6	0.32
**Post-traumatic stress disorder**							
Low	25	16 440	2 634	8.4 (4.7 – 13.1)	0.900	98.9	0.15
High	8	6 012	947	7.9 (2.2 – 12.2)		99.0	0.15
**Obsessive-compulsive disorder**							
Low	14	6 324	353	5.2 (1.5 – 10.9)	0.180	98.6	0.16
High	3	1 282	149	17.6 (2.4 – 42.4)		98.6	0.23
**Panic disorder**							
Low	11	4 637	186	4.0 (2.2 – 6.4)	0.060	91.7	0.03
High	2	1 091	23	2.1 (1.3 – 3.0)		0.02	0.00
**Social anxiety disorder**							
Low	6	2 504	134	5.3 (2.6 – 9.0)	--	90.0	0.03
High	0	0	0	--		--	--
**Adjustment disorder**							
Low	2	475	16	2.9 (0.0 – 14.1)	--	95.3	0.09
High	0	0	0	--		--	--

© 2023 Roddy Mitchell A et al. *JAMA Network Open.*

eReferences

1. Niazi AUR, Alekozay M, Osmani K, Najm AF. Prevalence and associated factors of depression, anxiety, and stress among pregnant women in Herat, Afghanistan: A cross-sectional study. Health Science Reports. 2023;6(8) (no pagination).
2. Begum MR, Biswas SC. Prevalence and associated factors of antenatal anxiety symptoms in Bangladesh: A repeated measures cluster data analysis. Psychiatria Danubina. 2021;33:52-7.
3. Edhborg M, Nasreen HE, Kabir ZN. Impact of postpartum depressive and anxiety symptoms on mothers' emotional tie to their infants 2-3 months postpartum: A population-based study from rural Bangladesh. Archives of Women's Mental Health. 2011;14(4):307-16.
4. Nasreen HE, Kabir ZN, Forsell Y, Edhborg M. Prevalence and associated factors of depressive and anxiety symptoms during pregnancy: a population based study in rural Bangladesh. BMC Women's Health. 2011;11:22.
5. Nasreen HE, Kabir ZN, Forsell Y, Edhborg M. Low birth weight in offspring of women with depressive and anxiety symptoms during pregnancy: results from a population based study in Bangladesh. BMC Public Health. 2010;10:515.
6. Lopes BCS, Lima CA, Ferreira TSB, de Freitas WML, Ferreira TB, de Pinho L, et al. Perceived stress and associated factors in pregnant women: a cross-sectional study nested within a population-based cohort. Revista Brasileira de Saude Materno Infantil. 2023;23 (no pagination).
7. Loret de Mola C, Carpena MX, Dias IM, Meucci R, Goicochea-Romero A, Cesar J. Sleep and its association with depressive and anxiety symptoms during the last weeks of pregnancy: A population-based study. Sleep health. 2023;9(4):482-8.
8. Scholl CC, Trettim JP, Bohm DM, Molina ML, Soares MC, Dias NDC, et al. Are adolescents more likely to have antenatal anxiety disorders than adult women? A comparison between two samples. Journal of Affective Disorders. 2022;316:50-5.
9. de Mello DB, Trettim JP, da Cunha GK, Rubin BB, Scholl CC, Ardais AP, et al. Generalized Anxiety Disorder, Depressive Symptoms and the Occurrence of Stressors Events in a Probabilistic Sample of Pregnant Women. Psychiatric Quarterly. 2021;92(1):123-33.
10. Farias DR, Carrilho TRB, Freitas-Costa NC, Batalha MA, Gonzalez M, Kac G. Maternal mental health and gestational weight gain in a Brazilian Cohort. Scientific reports. 2021;11(1):10787.
11. Muller EV, Martins CM, Borges PKO. Prevalence of anxiety and depression disorder and associated factors during postpartum in puerperal women. Revista Brasileira de Saude Materno Infantil. 2021;21(4):995-1004.
12. Nomura R, Tavares I, Ubinha AC, Costa ML, Opperman ML, Brock M, et al. Impact of the covid-19 pandemic on maternal anxiety in Brazil. Journal of Clinical Medicine. 2021;10(4):1-14.
13. Soares MC, de Matos MB, da Cunha GK, Leite CF, Caruccio HS, Trettim JP, et al. Suicide risk and prematurity: A study with pregnant adolescents. Journal of Psychiatric Research. 2021;133:125-33.
14. Pabon S, Parpinelli MA, Narvaez MB, Charles CMp, Guida JP, Escobar MF, et al. Overall Maternal Morbidity during Pregnancy Identified with the WHO-WOICE Instrument. BioMed Research International. 2020:1-9.
15. Costa DO, Souza FIS, Pedroso GC, Strufaldi MWL. Mental disorders in pregnancy and newborn conditions: longitudinal study with pregnant women attended in primary care. Ciencia & Saude Coletiva. 2018;23(3):691-700.
16. de Matos MB, Scholl CC, Trettim JP, Molina ML, Soares MC, Coelho FT, et al. The perception of parental bonding in pregnant adolescents and its association with generalized anxiety disorder. European Psychiatry: the Journal of the Association of European Psychiatrists. 2018;54:51-6.
17. Ferraro AA, Rohde LA, Polanczyk GV, Argeu A, Miguel EC, Grisi S, et al. The specific and combined role of domestic violence and mental health disorders during pregnancy on new-born health. BMC Pregnancy & Childbirth. 2017;17(1):257.
18. Mônica Maria de Jesus S, Denismar Alves N, Maria José C, Eliana Peres Rocha Carvalho L. Anxiety in pregnancy: prevalence and associated factors. Revista da Escola de Enfermagem da USP. 2017;51:1-8.

© 2023 Roddy Mitchell A et al. *JAMA Network Open.*

19. Paskulin JTA, Drehmer M, Olinto MT, Hoffmann JF, Pinheiro AP, Schmidt MI, et al. Association between dietary patterns and mental disorders in pregnant women in Southern Brazil. Revista Brasileira de Psiquiatria. 2017;39(3):208-15.
20. Peter PJ, de Mola CL, de Matos MB, Coelho FM, Pinheiro KA, da Silva RA, et al. Association between perceived social support and anxiety in pregnant adolescents. Revista Brasileira de Psiquiatria. 2017;39(1):21-7.
21. Castro ECT, Cardoso MN, Brancaglion MYM, Faria GC, Garcia FD, Nicolato R, et al. Antenatal depression: Prevalence and risk factor patterns across the gestational period. Journal of Affective Disorders. 2016;192:70-5.
22. Coelho FM, Pinheiro RT, Silva RA, de Avila Quevedo L, de Mattos Souza LD, de Matos MB, et al. Parental bonding and suicidality in pregnant teenagers: a population-based study in southern Brazil. Social Psychiatry & Psychiatric Epidemiology. 2014;49(8):1241-8.
23. Farias DR, Pinto Tde J, Teofilo MM, Vilela AA, Vaz Jdos S, Nardi AE, et al. Prevalence of psychiatric disorders in the first trimester of pregnancy and factors associated with current suicide risk. Psychiatry Research. 2013;210(3):962-8.
24. Takahasi EHM, e Alves MTSSB, Alves GS, da Silva AAM, Batista RFL, Simoes VMF, et al. Mental health and physical inactivity during pregnancy: A cross-sectional study nested in the BRISA cohort study. Cadernos de Saude Publica. 2013;29(8):1583-94.
25. da Silva RA, da Costa Ores L, Jansen K, da Silva Moraes IG, de Mattos Souza LD, Magalhaes P, et al. Suicidality and associated factors in pregnant women in Brazil. Community Mental Health Journal. 2012;48(3):392-5.
26. Pinheiro RT, da Cunha Coelho FM, da Silva RA, de Avila Quevedo L, de Mattos Souza LD, Castelli RD, et al. Suicidal behavior in pregnant teenagers in southern Brazil: social, obstetric and psychiatric correlates. Journal of Affective Disorders. 2012;136(3):520-5.
27. Tavares D, Quevedo L, Jansen K, Souza L, Pinheiro R, Silva R. Prevalence of suicide risk and comorbidities in postpartum women in Pelotas. Revista Brasileira de Psiquiatria. 2012;34(3):270-6.
28. Dias FMV, Junior CSD, Franco GC, Teixeira AL, Ribeiro AM. Pregnancy is associated with psychiatric symptoms in a low-income countryside community of Brazil. Neuropsychiatric Disease and Treatment. 2011;7:709-14.
29. Zambaldi CF, Cantilino A, Sougey EB. Bio-socio-demographic factors associated with post-traumatic stress disorder in a sample of postpartum Brazilian women. Archives of Women's Mental Health. 2011;14(5):435-9.
30. Silva CS, Ronzani TM, Furtado EF, Aliane PP, Moreira-Almeida A. Relationship between religious practice, alcohol use, and psychiatric disorders among pregnant women. Revista de Psiquiatria Clinica. 2010;37(4):152-6.
31. Zambaldi CF, Cantilino A, Montenegro AC, Paes JA, de Albuquerque TL, Sougey EB. Postpartum obsessive-compulsive disorder: prevalence and clinical characteristics. Comprehensive Psychiatry. 2009;50(6):503-9.
32. Faisal-Cury A, Rossi Menezes P. Prevalence of anxiety and depression during pregnancy in a private setting sample. Archives of Women's Mental Health. 2007;10(1):25-32.
33. Djatche Miafo J, Woks NIE, Nzebou D, Tchaptchet I, Delene ST, Kegha Tchidje O, et al. Epidemiological profile of perinatal mental disorders at a tertiary hospital in Yaounde- Cameroon. Frontiers in Global Womens Health. 2023;4:999840.
34. Chen W, Peng W, Zhang Y, Zhou H, Zhang M. Anxiety and depression among perinatal women during the long-term normal prevention of COVID-19 pandemic period in China: a cross-sectional study. BMC Psychiatry. 2023;23(1) (no pagination).
35. Gao Y, Tang X, Deng R, Liu J, Zhong X. Latent Trajectories and Risk Factors of Prenatal Stress, Anxiety, and Depression in Southwestern China-A Longitudinal Study. International Journal of Environmental Research and Public Health. 2023;20(5) (no pagination).
36. Hou Y, Shang M, Yu X, Gu Y, Li H, Lu M, et al. Joint effects of recent stressful life events and adverse childhood experiences on perinatal comorbid anxiety and depression. BMC Pregnancy and Childbirth. 2023;23(1) (no pagination).
37. Qin X, Zhang W, Xu S, Ma M, Fan X, Nie X, et al. Prevalence and risk factors of anxious and depressive symptoms in first-trimester females and their partners: a study during the pandemic era of COVID-19 in China. BMC Psychiatry. 2023;23(1) (no pagination).

© 2023 Roddy Mitchell A et al. *JAMA Network Open.*

38. Shi YY, Wei Q, Ma X, Zhang Y, Wang L, Shi HJ. Maternal affective and stress-related factors during pregnancy affect the occurrence of childhood allergic diseases: A Shanghai MCPC study. Journal of Psychosomatic Research.165:111142.
39. Yang HD, Pan YY, Chen WM, Yang X, Liu B, Yuan N, et al. Prevalence of and relevant factors for depression and anxiety symptoms among pregnant women on the eastern seaboard of China in the post-COVID-19 era: a cross-sectional study. Bmc Psychiatry. 2023;23(1).
40. Yang JP, Qu J, Sun K, Gao LL. Anxiety symptoms and health-related quality of life in mainland Chinese pregnant women: a cross-sectional study. Journal of Reproductive and Infant Psychology. 2023;41(1):3-14.
41. Yang J, Lin XZ, Guo QW, Wang CL, Yang RY, Zhang JW, et al. Mediating effect of mindfulness level on the relationship between marital quality and postpartum depression among primiparas. World Journal of Clinical Cases. 2023;11(12):2729-39.
42. Zhang X, Ma P, Li M. The association between paternal childcare involvement and postpartum depression and anxiety among Chinese women-a path model analysis. Archives of Women's Mental Health. 2023;26(1):99-106.
43. Zhou X, Rao L, Yang D, Wang T, Li H, Liu Z. Effects of maternal pre-pregnancy body mass index and gestational weight gain on antenatal mental disorders in China: a prospective study. BMC Pregnancy Childbirth. 2023;23(1):188.
44. Chen H, Zou Y, Shi H, Ma H, Huang W, Wang S, et al. COVID-19 affects psychological symptoms of pregnant women indirectly by increasing their maternal concerns. Journal of Affective Disorders. 2022;317:79-83.
45. Jigeer G, Tao W, Zhu Q, Xu X, Zhao Y, Kan H, et al. Association of residential noise exposure with maternal anxiety and depression in late pregnancy. Environment International. 2022;168 (no pagination).
46. Ma S, Yin X, Tao R, Jiang X, Xie J, Li P, et al. Association of maternal prenatal depression and anxiety with toddler sleep: The China-Anhui birth cohort study. Archives of Women's Mental Health. 2022;25(2):431-9.
47. Pages N, Gorgui J, Wang C, Wang X, Zhao JP, Tchuente V, et al. The Impact of COVID-19 on Maternal Mental Health during Pregnancy: A Comparison between Canada and China within the CONCEPTION Cohort. International Journal of Environmental Research and Public Health. 2022;19(19) (no pagination).
48. Wang L, Yang N, Zhou H, Mao X, Zhou Y. Pregnant Women's Anxiety and Depression Symptoms and Influence Factors in the COVID-19 Pandemic in Changzhou, China. Frontiers in Psychology.13:855545.
49. Wu F, Zhou L, Chen C, Lin W, Liu P, Huang W, et al. Association between intimate partner violence and prenatal anxiety and depression in pregnant women: A cross-sectional survey during the COVID-19 epidemic in Shenzhen, China. BMJ Open. 2022;12(5) (no pagination).
50. Yang JP, Lin RJ, Sun K, Gao LL. Incidence and correlates of insomnia and its impact on health-related quality of life among Chinese pregnant women: a cross-sectional study. Journal of Reproductive and Infant Psychology. 2022.
51. Zhang J, Yuan H, Xu L, Yi C, Tang W. The impact of COVID-19 on the mental health of pregnant women in Shanghai, China. Frontiers in public health. 2022;10:938156.
52. Zhou Y, Huang J, Baker PN, Liao B, Yu X. The prevalence and associated factors of prenatal depression and anxiety in twin pregnancy: a cross-sectional study in Chongqing, China. BMC Pregnancy and Childbirth. 2022;22(1) (no pagination).
53. Cao Y, Liu J, Zhang Y, Li Y, Chen Z, Lu J. Pregnant women's psychological state and influence factors: anxiety, and depression during COVID-19 outbreak. Journal of Perinatal Medicine.49(6):664-73.
54. Cui C, Zhai L, Sznajder KK, Wang J, Sun X, Wang X, et al. Prenatal anxiety and the associated factors among Chinese pregnant women during the COVID-19 pandemic--a smartphone questionnaire survey study. BMC Psychiatry. 2021;21(1) (no pagination).
55. Ding W, Lu J, Zhou Y, Wei W, Zhou Z, Chen M. Knowledge, attitudes, practices, and influencing factors of anxiety among pregnant women in Wuhan during the outbreak of COVID-19: a cross-sectional study. BMC Pregnancy & Childbirth. 2021;21(1):80.

© 2023 Roddy Mitchell A et al. *JAMA Network Open.*

56. Dong H, Hu R, Lu C, Huang D, Cui D, Huang G, et al. Investigation on the mental health status of pregnant women in China during the Pandemic of COVID-19. Archives of Gynecology and Obstetrics. 2021;303(2):463-9.
57. Ge Y, Shi C, Wu B, Liu Y, Chen L, Deng Y. Anxiety and adaptation of behavior in pregnant Zhuang women during the COVID-19 pandemic: A mixed-mode survey. Risk Management and Healthcare Policy. 2021;14:1563-73.
58. Guo J, Zheng A, He J, Ai M, Gan Y, Zhang Q, et al. The prevalence of and factors associated with antenatal depression among all pregnant women first attending antenatal care: a cross-sectional study in a comprehensive teaching hospital. BMC Pregnancy and Childbirth. 2021;21(1) (no pagination).
59. Jiang Q, Guo Y, Zhang E, Cohen N, Ohtori M, Sun A, et al. Perinatal Mental Health Problems in Rural China: The Role of Social Factors. Frontiers in Psychiatry. 2021;12 (no pagination).
60. Jiang H, Jin L, Qian X, Xiong X, La X, Chen W, et al. The mental health status and approaches of accessing antenatal care information among pregnant women during COVID-19 epidemic ：a cross-sectional study in China. Journal of Medical Internet Research. 2021;23(1):N.PAG-N.PAG.
61. Lin W, Wu B, Chen B, Lai G, Huang S, Li S, et al. Sleep Conditions Associate with Anxiety and Depression Symptoms among Pregnant Women during the Epidemic of COVID-19 in Shenzhen. Journal of Affective Disorders. 2021;281:567-73.
62. Liu M, Li N, Cai X, Feng X, Wang R, Xiong P. The Prevalence of Psychological Symptoms in Pregnant Healthcare Workers (HCWs) and Pregnant Non-HCWs During the Early Stage of COVID-19 Pandemic in Chongqing, China. Frontiers in psychiatry Frontiers Research Foundation. 2021;12:708698.
63. Luo Z, Xue L, Ma L, Liu Z. Comorbid Anxiety and Depression and Related Factors Among Pregnant and Postpartum Chinese Women During the Coronavirus Disease 2019 Pandemic. Frontiers in Psychology. 2021;12:701629.
64. Ma R, Yang F, Zhang L, Sznajder KK, Zou C, Jia Y, et al. Resilience mediates the effect of self-efficacy on symptoms of prenatal anxiety among pregnant women: a nationwide smartphone cross-sectional study in China. BMC Pregnancy and Childbirth. 2021;21(1) (no pagination).
65. Mo PKH, Fong VWI, Song B, Di J, Wang Q, Wang L. Association of Perceived Threat, Negative Emotions, and Self-Efficacy With Mental Health and Personal Protective Behavior Among Chinese Pregnant Women During the COVID-19 Pandemic: Cross-sectional Survey Study. Journal of Medical Internet Research. 2021;23(4):e24053.
66. Peng S, Zhang Y, Liu H, Huang X, Noble DJ, Yang L, et al. A multi-center survey on the postpartum mental health of mothers and attachment to their neonates during COVID-19 in Hubei Province of China. Annals of Translational Medicine. 2021;9(5) (no pagination).
67. Wang Q, Mo PKH, Song B, Di JL, Zhou FR, Zhao J, et al. Mental health and preventive behaviour of pregnant women in China during the early phase of the COVID-19 period. Infect Dis Poverty. 2021;10(1):37.
68. Xie M, Wang X, Zhang J, Wang Y. Alteration in the psychologic status and family environment of pregnant women before and during the COVID-19 pandemic. International Journal of Gynaecology & Obstetrics. 2021;153(1):71-5.
69. Xu K, Zhang Y, Xu Q, Lv L, Zhang J. Mental health among pregnant women under public health interventions during COVID-19 outbreak in Wuhan, China. Psychiatry Research. 2021;301 (no pagination).
70. Yang X, Song B, Wu ANS, Mo PKH, Di JL, Wang Q, et al. Social, Cognitive, and eHealth Mechanisms of COVID-19-Related Lockdown and Mandatory Quarantine That Potentially Affect the Mental Health of Pregnant Women in China: Cross-Sectional Survey Study. Journal of Medical Internet Research. 2021;23(1).
71. Zhang CJP, Wu H, He Z, Chan NK, Huang J, Wang H, et al. Psychobehavioral Responses, Post-Traumatic Stress and Depression in Pregnancy During the Early Phase of COVID-19 Outbreak. Psychiatric Research and Clinical Practice. 2021;3(1):46-54.
72. Zhang Y, Zhang Y, Deng R, Chen M, Cao R, Chen S, et al. Association of Sleep Duration and Screen Time With Anxiety of Pregnant Women During the COVID-19 Pandemic. Frontiers in Psychology. 2021;12:646368.

© 2023 Roddy Mitchell A et al. *JAMA Network Open.*

73. Liu Y, Guo N, Li T, Zhuang W, Jiang H. Prevalence and Associated Factors of Postpartum Anxiety and Depression Symptoms Among Women in Shanghai, China. Journal of Affective Disorders. 2020;274:848-56.
74. Liu X, Chen M, Wang Y, Sun L, Zhang J, Shi Y, et al. Prenatal anxiety and obstetric decisions among pregnant women in Wuhan and Chongqing during the COVID-19 outbreak: a cross-sectional study. BJOG: An International Journal of Obstetrics & Gynaecology. 2020;127(10):1229-40.
75. Lu L, Duan Z, Wang Y, Wilson A, Yang Y, Zhu L, et al. Mental health outcomes among Chinese prenatal and postpartum women after the implementation of universal two-child policy. Journal of Affective Disorders. 2020;264:187-92.
76. Yu YH, Zhu XD, Xu HL, Hu Z, Zhou WS, Zheng BH, et al. Prevalence of depression symptoms and its influencing factors among pregnant women in late pregnancy in urban areas of Hengyang City, Hunan Province, China: a cross-sectional study. Bmj Open. 2020;10(9).
77. Yue C, Liu C, Wang J, Zhang M, Wu H, Li C, et al. Association between social support and anxiety among pregnant women in the third trimester during the coronavirus disease 2019 (COVID-19) epidemic in Qingdao, China: The mediating effect of risk perception. The International journal of social psychiatry. 2021;67(2):120-7.
78. Zeng XQ, Li WG, Sun HW, Luo X, Garg S, Liu T, et al. Mental Health Outcomes in Perinatal Women During the Remission Phase of COVID-19 in China. Frontiers in Psychiatry. 2020;11.
79. Zhou Y, Shi H, Liu Z, Peng S, Wang R, Qi L, et al. The prevalence of psychiatric symptoms of pregnant and non-pregnant women during the COVID-19 epidemic. Transl Psychiatry Psychiatry. 2020;10(1):319.
80. Duan Z, Wang Y, Tao Y, Bower JL, Yu R, Wang S, et al. Relationship between trait neuroticism and suicidal ideation among postpartum women in China: Testing a mediation model. Journal of Affective Disorders. 2019;256:532-5.
81. Ma X, Wang Y, Hu H, Tao XG, Zhang Y, Shi H. The impact of resilience on prenatal anxiety and depression among pregnant women in Shanghai. Journal of Affective Disorders. 2019;250:57-64.
82. Tang X, Lu Z, Hu DH, Zhong XN. Influencing factors for prenatal Stress, anxiety and depression in early pregnancy among women in Chongqing, China. Journal of Affective Disorders. 2019;253:292-302.
83. Zhang Y, Muyiduli X, Wang S, Jiang W, Wu J, Li M, et al. Prevalence and relevant factors of anxiety and depression among pregnant women in a cohort study from south-east China. Journal of Reproductive and Infant Psychology. 2018.
84. Yu Y, Li M, Pu L, Wang S, Wu J, Ruan L, et al. Sleep was associated with depression and anxiety status during pregnancy: a prospective longitudinal study. Archives of Women's Mental Health. 2017;20(5):695-701.
85. Zeng Y, Li Y, Xia H, Wang S, Zhou J, Chen D. Retinoids, anxiety and peripartum depressive symptoms among Chinese women: a prospective cohort study. BMC Psychiatry. 2017;17(1):278.
86. Kang YT, Yao Y, Dou J, Guo X, Li SY, Zhao CN, et al. Prevalence and Risk Factors of Maternal Anxiety in Late Pregnancy in China. International Journal of Environmental Research & Public Health [Electronic Resource]. 2016;13(5):04.
87. Qu Z, Wang X, Tian D, Zhao Y, Zhang Q, He H, et al. Posttraumatic stress disorder and depression among new mothers at 8 months later of the 2008 Sichuan earthquake in China. Archives of Women's Mental Health. 2012;15(1):49-55.
88. Qu Z, Tian D, Zhang Q, Wang X, He H, Zhang X, et al. The impact of the catastrophic earthquake in China's Sichuan province on the mental health of pregnant women. Journal of Affective Disorders. 2012;136(1/2):117-23.
89. Qiao YX, Wang J, Li J, Ablat A. The prevalence and related risk factors of anxiety and depression symptoms among Chinese pregnant women in Shanghai. Australian & New Zealand Journal of Obstetrics & Gynaecology. 2009;49(2):185-90.
90. Guillen-Burgos HF, Galvez-Florez JF, Miranda J, Hincapie-Porras C, Perez-Olivo JL, Piraquive-Caicedo JP, et al. Pregnancy and mental health outcomes during the COVID-19 pandemic in Colombia: A nationwide cross-sectional study. Journal of Affective Disorders Reports. 2023;12 (no pagination).
91. Esquivel Lauzurique M, Vera Fernandez Y, Dennis CL, Ruben Quesada M, Alvarez Valdes G, Lye S, et al. Prevalence, Incidence, and Persistence of Postpartum Anxiety, Depression, and Comorbidity: A

Cohort Study Among Women in Havana Cuba. Journal of Perinatal and Neonatal Nursing. 2022;36(4):E15-E24.
92. Ahmed GK, Salman SA, Elbeh K, Amer ZS, Abbas AM. Correlation between psychiatric impact of COVID-19 during pregnancy and fetal outcomes in Egyptian women. Psychiatry Research. 2022;317 (no pagination).
93. Wassif OM, Abdo AS, Elawady MA, Abd Elmaksoud AE, Eldesouky RS. Assessment of Postpartum Depression and Anxiety among Females Attending Primary Health Care Facilities in Qaliubeya Governorate, Egypt. Journal of Environmental and Public Health. 2019;2019 (no pagination).
94. Abdelhai R, Mosleh H. Screening for antepartum anxiety and depression and their association with domestic violence among Egyptian pregnant women. Journal of the Egyptian Public Health Association. 2015;90(3):101-8.
95. Malaju MT. A structural equation modelling of the direct and indirect factors associated with functional status over time as measured by WHODAS-32 items among postpartum women in Northwest Ethiopia. Archives of Public Health. 2023;81(1):41.
96. Tibebu NS, Kassie BA, Anteneh TA, Rade BK. Depression, anxiety and stress among HIV-positive pregnant women in Ethiopia during the COVID-19 pandemic. Transactions of the Royal Society of Tropical Medicine and Hygiene. 2023;117(5):317-25.
97. Bishaw KA, Andalem A, Amha H, Wondie T. Generalized Anxiety Disorder and Its Associated Factors Among Pregnant Women During COVID-19 at Public Health Facilities of East Gojjam Zone, 2020: A Multi-Center Cross-Sectional Study. Frontiers in Global Womens Health.3:918332.
98. Lelisho ME, Merera AM, Tareke SA, Hassen SS, Jemal SS, Markos Kontuab A, et al. Generalized anxiety disorder among mothers attending perinatal services during COVID-19 pandemic: using ordinal logistic regression model. Heliyon.8(6):e09778.
99. Bante A, Mersha A, Zerdo Z, Wassihun B, Yeheyis T. Comorbid anxiety and depression: Prevalence and associated factors among pregnant women in Arba Minch zuria district, Gamo zone, southern Ethiopia. PLoS ONE [Electronic Resource]. 2021;16(3):e0248331.
100. Beketie ED, Kahsay HB, Nigussie FG, Tafese WT. Magnitude and associated factors of antenatal depression among mothers attending antenatal care in Arba Minch town, Ethiopia, 2018. PLoS ONE. 2021;16(12 December) (no pagination).
101. Kassaw C, Pandey D. The prevalence of general anxiety disorder and its associated factors among women's attending at the perinatal service of Dilla University referral hospital, Dilla town, Ethiopia, April, 2020 in Covid pandemic. Heliyon. 2020;6(11):e05593.
102. Kugbey N, Ayanore M, Doegah P, Chirwa M, Bartels SA, Davison CM, et al. Prevalence and Correlates of Prenatal Depression, Anxiety and Suicidal Behaviours in the Volta Region of Ghana. International Journal of Environmental Research & Public Health [Electronic Resource]. 2021;18(11):29.
103. Ryali S, Kumar MS, Ryali V, Paspulati S. Is cesarean section a clinical marker for psychiatric and sleep disorder in young mothers? A cross-sectional study from rural South India. Industrial Psychiatry Journal.32(1):158-63.
104. Bachani S, Sahoo SM, Nagendrappa S, Dabral A, Chandra P. Anxiety and depression among women with COVID-19 infection during childbirth-experience from a tertiary care academic center. AJOG Global Reports. 2022;2(1) (no pagination).
105. Nazir T, Amin R, Maqbool M. Emotional difficulties in pregnant females who tested positive for COVID-19: A cross-sectional study from South Kashmir, India. Journal of Education & Health Promotion.11:13.
106. Tiwari M, Pise, H.N., & TIWARI, M. Generalized anxiety disorderand factors affecting it during the postnatal period: an observational study. Asian Journal of Pharmaceutical and Clinical Research. 2022;15(5):90-3.
107. Ray PB, Chakraborty MK, Hazra S. A Cross-sectional Study to Assess the Anxiety and Depression among Perinatal Mothers during the COVID-19 Pandemic. Journal of SAFOG. 2022;14(2):106-10.
108. Singh B, Devalla A, Pushpalatha K, Gautam N, Dabar D. Knowledge and Anxiety of Pregnant Women towards COVID-19 Pandemic in the Prevaccination Phase. Journal of Clinical and Diagnostic Research. 2022;16(6):QC01-QC5.

© 2023 Roddy Mitchell A et al. *JAMA Network Open.*

109. Thurkkada AP, Joseph, N. E., Manoj, G., & Ravindran, G.C. Prenatal Anxiety, Perceived Stress, and Coping Behaviour regarding Covid-19 among Pregnant Women at a selected Hospital, Kochi, South India. Africa Journal of Nursing & Midwifery. 2022;24(2):1-10.
110. Jelly P, Chadha L, Kaur N, Sharma S, Sharma R, Stephen S, et al. Impact of COVID-19 Pandemic on the Psychological Status of Pregnant Women. Cureus. 2021;13(1).
111. Jha S, Salve HR, Goswami K, Sagar R, Kant S. Prevalence of Common Mental Disorders among pregnant women-Evidence from population-based study in rural Haryana, India. Journal of Family Medicine & Primary Care.10(6):2319-24.
112. Maria C, Ramesh N, Johnson AR, Prince PM, Rodrigues A, Lekha A, et al. Prevalence and Determinants of Postpartum Anxiety among Women Availing Health Services at a Rural Maternity Hospital in South India. Journal of SAFOG. 2021;13(1):1-5.
113. Tikka SK, Parial S, Pattojoshi A, Bagadia A, Prakash C, Lahiri D, et al. Anxiety among pregnant women during the COVID-19 pandemic in India - A multicentric study. Asian Journal of Psychiatry. 2021;66 (no pagination).
114. Ture P, Dambhare DG, Mundra A, Raut AV, Maliye CH, Deshmukh PR, et al. Magnitude and determinants of psychological morbidities among pregnant women: Results from a pregnancy cohort in rural Central India. Medical Journal Armed Forces India. 2022.
115. Bhushan NL, Krupp K, Jaykrishna P, Ravi K, Khan A, Shidhaye R, et al. The association between social support through contacts with Accredited Social Health Activists (ASHAs) and antenatal anxiety among women in Mysore, India: a cross-sectional study. Social Psychiatry and Psychiatric Epidemiology. 2020;55(10):1323-33.
116. Goyal S, Gupta B, Sharma E, Dalal PK, Pradeep Y. Psychiatric Morbidity, Cultural Factors, and Health-Seeking Behaviour in Perinatal Women: A Cross-Sectional Study from a Tertiary Care Centre of North India. Indian Journal of Psychological Medicine. 2020;42(1):52-60.
117. Kantipudi SJ, Kannan GK, Viswanathan S, Ranganathan S, Menon J, Ramanathan S. Antenatal Depression and Generalized Anxiety Disorder in a Tertiary Hospital in South India. Indian Journal of Psychological Medicine. 2020;42(6):513-8.
118. Khatri S, Murthy AK, Hashim U, Kuruthukulangara S, Kumari A, Lele PR. Psychological status of pregnant women during COVID-19 pandemic: A cross-sectional study from Mumbai. Journal of Marine Medical Society. 2020;22(3):113-7.
119. Boggaram SA, Singh H, Manikanta TS, Maheswari E. An exploratory study of identification of psychiatric disorders during pregnancy. Minerva Psichiatrica. 2017;58(4):203-8.
120. Margawati A, Syauqy A, Utami A, Hananingtyas A, Zaimatussoleha C. Anxiety among Pregnant Women in Rural-Urban Area Indonesia during the COVID-19 Pandemic in Semarang, Indonesia. Open Access Macedonian Journal of Medical Sciences. 2022;10(E):1830-7.
121. Zainiyah Z, Susanti E. Anxiety in Pregnant Women During Coronavirus (Covid-19) Pandemic in East Java, Indonesia. Majalah Kedokteran Bandung-Mkb-Bandung Medical Journal. 2020;52(3):149-53.
122. Maleki A, Ashtari M, Molaie P, Youseflu S. Influential factors of general anxiety disorder among Iranian pregnant women during the second peak of COVID-19 pandemic. Psychology, health & medicine. 2022;27(2):421-7.
123. Keramat A, Malary M, Moosazadeh M, Bagherian N, Rajabi-Shakib MR. Factors influencing stress, anxiety, and depression among Iranian pregnant women: the role of sexual distress and genital self-image. BMC Pregnancy & Childbirth.21(1):87.
124. Maharlouei N, Keshavarz P, Salemi N, Lankarani KB. Depression and anxiety among pregnant mothers in the initial stage of the Coronavirus Disease (COVID-19) pandemic in the southwest of Iran. Reproductive Health. 2021;18(1) (no pagination).
125. Effati-Daryani F, Zarei S, Mohammadi A, Hemmati E, Ghasemi Yngyknd S, Mirghafourvand M. Depression, stress, anxiety and their predictors in Iranian pregnant women during the outbreak of COVID-19. BMC psychology. 2020;8(1):99.
126. Youseflu S, Bayat Z, Amiri F, Mohebbi P. The role of reproductive variables, anxiety, physical activity, on the sleep quality of lactating women referring to health care centers of Zanjan-Iran. Nursing & Midwifery Care Journal. 2020;10(1):39-45.
127. Alipour Z, Kheirabadi GR, Eslami AA, Kazemi A. Psychological profiles of risk for antenatal depression and anxiety in Iranian sociocultural context. Journal of Education & Health Promotion. 2018;7:160.

© 2023 Roddy Mitchell A et al. *JAMA Network Open.*

128. Mahmoodi H, Golboni F, Nadrian H, Zareipour M, Shirzadi S, Gheshlagh RG. Mother-Father Differences in Postnatal Psychological Distress and Its Determinants in Iran. Open Access Macedonian Journal of Medical Sciences. 2017;5(1):91-6.
129. Mahmoodi Z, Dolatian M, Shaban Z, Shams J, Alavi-Majd H, Mirabzadeh A. Correlation between Kind of Delivery and Posttraumatic Stress Disorder. Annals of Medical and Health Sciences Research. 2016;6(6):356-61.
130. Shaban Z, Dolatian M, Shams J, Alavi-Majd H, Mahmoodi Z, Sajjadi H. Post-Traumatic Stress Disorder (PTSD) Following Childbirth: Prevalence and Contributing Factors. Iranian Red Crescent Medical Journal. 2013;15(3):177-82.
131. Bataineh MF, Mohamad MN, Al Dhaheri AS, Rawashdeh M, Al-Nawaiseh AM, Asali FF, et al. Impact of coronavirus 2019 pandemic on post-traumatic stress disorder symptoms among pregnant women in Jordan. Women's Health. 2022;18(no pagination).
132. Basha AS, Sabanekh CS, Shlash LK, Dawod LN, Dweik MM, Obeidat MN, et al. Prevalence of anxiety among pregnant women attending antenatal care in jordan: A single center study. Jordan Journal of Pharmaceutical Sciences. 2021;14(3):351-8.
133. Gerges S, Obeid S, Hallit S. Traversing mental health disorders during pregnancy: Lebanese women's experiences of antepartum depression and anxiety. Irish journal of medical science. 2023;20.
134. Nasreen HE, Pasi HB, Aris MAM, Rahman JA, Rus RM, Edhborg M. Impact of parental perinatal depressive and anxiety symptoms trajectories on early parent-infant impaired bonding: a cohort study in east and west coasts of Malaysia. Archives of Women's Mental Health. 2022;25(2):377-87.
135. Kalok A, Syed Anwar Aly SA, Abdul Rahman R, Mahdy ZA, Sharip S. COVID-19 Pandemic and Maternal Psychological Wellbeing During the Malaysian Movement Control Order: A Cross-Sectional Study. Frontiers in Psychiatry. 2022;12 (no pagination).
136. Azlan WAW, Ramalingam M, Razali R, Abdullah MF, Rahman FNA. Anxiety, depression and marital satisfaction in women with hyperemesis gravidarum: A comparative cross-sectional study in Hospital Tengku Ampuan Rahimah, Klang, Malaysia. Asia-Pacific psychiatry : Official Journal of the Pacific Rim College of Psychiatrists.14(1):e12416.
137. Nasreen HE, Rahman JA, Rus RM, Kartiwi M, Sutan R, Edhborg M. Prevalence and determinants of antepartum depressive and anxiety symptoms in expectant mothers and fathers: Results from a perinatal psychiatric morbidity cohort study in the east and west coasts of Malaysia. BMC Psychiatry. 2018;18(1).
138. Jusoh ASB, Abdullah KL, Ahmad AB, Ghazali SB, Shafie ZBM, Mansor MB, et al. Anxiety Symptoms and Associated Factors among Outpatient Antenatal Mother: A Cross Sectional Study at University Malaya Medical Centre, Malaysia. International Medical Journal. 2014;21(6):531-5.
139. Fadzil A, Balakrishnan K, Razali R, Sidi H, Malapan T, Japaraj RP, et al. Risk factors for depression and anxiety among pregnant women in Hospital Tuanku Bainun, Ipoh, Malaysia. Asia-Pacific Psychiatry. 2013;5(SUPPL. 1):7-13.
140. Padilla JJ, Lara-Cinisomo S, Navarrete L, Lara MA. Perinatal Anxiety Symptoms: Rates and Risk Factors in Mexican Women. International Journal of Environmental Research and Public Health. 2021;18(1).
141. Hababa H, Assarag B. Measurement of maternal morbidity during postpartum with the WHO-WOICE tools in Morocco. BMC Pregnancy and Childbirth. 2023;23(1) (no pagination).
142. Khan S, Scorza P, Lovero KL, Dos Santos P, Fumo W, Camara B, et al. Women's mental health in Mozambique: is maternity a protective factor? Global Mental Health. 2022;9:38-44.
143. Mateus V, Cruz S, Costa R, Mesquita A, Christoforou A, Wilson CA, et al. Rates of depressive and anxiety symptoms in the perinatal period during the COVID-19 pandemic: Comparisons between countries and with pre-pandemic data. Journal of Affective Disorders. 2022;316:245-53.
144. Qasrawi R, Amro M, VicunaPolo S, Abu Al-Halawa D, Agha H, Abu Seir R, et al. Machine learning techniques for predicting depression and anxiety in pregnant and postpartum women during the COVID-19 pandemic: A cross-sectional regional study. F1000Research. 2022;11 (no pagination).
145. Bindt C, Guo N, Te Bonle M, Appiah-Poku J, Hinz R, Barthel D, et al. No association between antenatal common mental disorders in low-obstetric risk women and adverse birth outcomes in their offspring: Results from the CDS study in Ghana and Cote D'Ivoire. PLoS ONE. 2013;8(11).

© 2023 Roddy Mitchell A et al. *JAMA Network Open.*

146. Bindt C, Appiah-Poku J, Te Bonle M, Schoppen S, Feldt T, Barkmann C, et al. Antepartum depression and anxiety associated with disability in African women: cross-sectional results from the CDS study in Ghana and Cote d'Ivoire. PLoS ONE [Electronic Resource]. 2012;7(10):e48396.
147. Shrestha D, Saha R, Manandhar N, Adhikari A, Dahal J. Anxiety among pregnant women about corona virus infections during covid-19 pandemic at a tertiary care center in nepal: A descriptive cross-sectional study. Journal of the Nepal Medical Association. 2021;59(234):152-5.
148. Aryal KK, Alvik A, Thapa N, Mehata S, Roka T, Thapa P, et al. Anxiety and Depression among Pregnant Women and Mothers of Children Under one Year in Sindupalchowk District. Journal of Nepal Health Research Council. 2018;16(2):195-204.
149. Shrestha S, Adachi K, Petrini MA, Shrestha S. Factors associated with post-natal anxiety among primiparous mothers in Nepal. International Nursing Review. 2014;61(3):427-34.
150. Verbeek T, Arjadi R, Vendrik JJ, Burger H, Berger MY. Anxiety and depression during pregnancy in Central America: a cross-sectional study among pregnant women in the developing country Nicaragua. BMC Psychiatry. 2015;15:292.
151. Eleje GU, Oguejiofor CB, Oriji SO, Ekwuazi KE, Ugwu EO, Igbodike EP, et al. Depression, anxiety, and stress and adverse pregnancy outcomes in pregnant women with history of recurrent pregnancy loss in Nigeria. International Journal of Psychiatry in Medicine.912174231199215.
152. Wegbom AI, Edet CK, Ogba AA, Osaro BO, Harry AM, Pepple BG, et al. Determinants of Depression, Anxiety, and Stress among Pregnant Women Attending Tertiary Hospitals in Urban Centers, Nigeria. Women (2673-4184). 2023;3(1):41-52.
153. Ade-Ojo IP, Dada MU, Adeyanju TB. Comparison of Anxiety and Depression Among HIV-Positive and HIV-Negative Pregnant Women During COVID-19 Pandemic in Ekiti State, Southwest Nigeria. International Journal of General Medicine. 2022;15:4123-30.
154. Nwafor JI, Okedo-Alex IN, Ikeotuonye AC. Prevalence and predictors of depression, anxiety, and stress symptoms among pregnant women during COVID-19-related lockdown in Abakaliki, Nigeria. Malawi medical journal : the journal of Medical Association of Malawi. 2021;33(1):54-8.
155. Agbaje OS, Anyanwu JI, Umoke PIC, Iwuagwu TE, Iweama CN, Ozoemena EL, et al. Depressive and anxiety symptoms and associated factors among postnatal women in Enugu-North Senatorial District, South-East Nigeria: a cross-sectional study. Archives of Public Health. 2019;77:1.
156. Odinka P, Odinka J, Ezeme M, Ndukuba A, Amadi K, Muomah R, et al. Socio-demographic correlates of postpartum psychological distress among apparently healthy mothers in two tertiary hospitals in Enugu, South-East Nigeria. African Health Sciences. 2019;19(3):2515-25.
157. Adewuya AO, Ola BA, Aloba OO, Mapayi BM. Anxiety disorders among Nigerian women in late pregnancy: a controlled study. Archives of Women's Mental Health. 2006;9(6):325-8.
158. Abiodun OA, Adetoro OO, Ogunbode OO. Psychiatric morbidity in a pregnant population in Nigeria. General Hospital Psychiatry. 1993;15(2):125-8.
159. Aderibigbe YA, Gureje O. The validity of the 28-item General Health Questionnaire in a Nigerian antenatal clinic. Social Psychiatry & Psychiatric Epidemiology. 1992;27(6):280-3.
160. Lalani S, Premji SS, Shaikh K, Sulaiman S, Yim IS, Forcheh N, et al. Individual and collective contribution of antenatal psychosocial distress conditions and preterm birth in Pakistani women. PLoS ONE. 2023;18(3 March) (no pagination).
161. Ahmed M, Amin F, Taj A, Durrani N. Antenatal anxiety and depression: Frequency and correlates during the COVID-19 pandemic in Pakistan. Journal of Family Medicine & Primary Care.11(10):6407-15.
162. Irum S, Khan AA, Rabbani U, Lodhi FS, Elsous A. Frequency and Risk Factors of Anxiety and Depression among Pregnant Women in Abbottabad, Pakistan: A Facility-Based Cross-Sectional Study. Pakistan Journal of Medical and Health Sciences. 2022;16(7):465-8.
163. Gul E, Muneeb PM, Azeemi MU, Khan MA, Shah S. Antenatal anxiety and depression among pregnant women attending tertiary care hospital, Mardan, Pakistan. Khyber Medical University Journal-Kmuj. 2019;11(3):160-4.
164. Naseer MH, Hussain J, Yaqub HMH. Occurrence of psychiatric Disorders Among Pregnant Females. Indo American Journal of Pharmaceutical Sciences. 2019;6(6):11522-5.
165. Shagufta S, Shams S. Prevalence, Differences, and Predictors of Anxiety and Depression among Pregnant and Non-Pregnant Women in Peshawar Khyber Pakhtunkhwa Pakistan. Fwu Journal of Social Sciences. 2019;13(1):167-76.

© 2023 Roddy Mitchell A et al. *JAMA Network Open.*

166. Shehroz M, Kazmi A, Manzoor S. Depression and anxiety during pregnancy period. Indo American Journal of Pharmaceutical Sciences. 2019;6(5):9121-4.
167. Tariq M, Fatima S, Akhtar N, Bajwa SMA, Huma S, Shade MN. To Determine the Frequency of Panic Disorders and Social Phobia in Teenage Pregnancy Presenting in a Tertiary Hospital in Pakistan. Pakistan Journal of Medical & Health Sciences. 2019;13(3):682-4.
168. Waqas A, Raza N, Lodhi HW, Muhammad Z, Jamal M, Rehman A. Psychosocial Factors of Antenatal Anxiety and Depression in Pakistan: Is Social Support a Mediator? Plos One. 2015;10(1).
169. Ali NS, Azam IS, Ali BS, Tabbusum G, Moin SS. Frequency and associated factors for anxiety and depression in pregnant women: a hospital-based cross-sectional study. Thescientificworldjournal. 2012;2012:653098.
170. Niaz S, Izhar N, Bhatti MR. Anxiety and depression in pregnant women presenting in the OPD of a teaching hospital. Pakistan Journal of Medical Sciences. 2004;20(2):117-9.
171. Gelaye B, Sanchez SE, Andrade A, Gomez O, Coker AL, Dole N, et al. Association of antepartum depression, generalized anxiety, and posttraumatic stress disorder with infant birth weight and gestational age at delivery. Journal of Affective Disorders. 2020;262:310-6.
172. Umuziga MP, Adejumo O, Hynie M. A cross-sectional study of the prevalence and factors associated with symptoms of perinatal depression and anxiety in Rwanda. BMC Pregnancy & Childbirth. 2020;20(1):68.
173. Mare K, Pellowski, J., Koopwitz, S., Hoffman, N., van der Westhuizen, C., Wokman, L., & Stien, D. Perinatal suicidality: prevalence and correlates in a South African birth cohort. Archives of Women's Mental Health. 2021;24:737–48.
174. Redinger S, Pearson RM, Houle B, Norris SA, Rochat TJ. Antenatal depression and anxiety across pregnancy in urban South Africa. Journal of Affective Disorders. 2020;277:296-305.
175. Malemela RD, Mashegoane S. The Prevalence of Obsessive-Compulsive Disorder Symptoms and their Psychological Correlates amongst Pregnant Clinic Attendees in the Capricorn District, South Africa. African Journal of Reproductive Health. 2019;23(2):44-55.
176. Abrahams Z, Lund C, Field S, Honikman S. Factors associated with household food insecurity and depression in pregnant South African women from a low socio-economic setting: a cross-sectional study. Social Psychiatry and Psychiatric Epidemiology. 2018;53(4):363-72.
177. Redinger S, Norris SA, Pearson RM, Richter L, Rochat T. First trimester antenatal depression and anxiety: prevalence and associated factors in an urban population in Soweto, South Africa. Journal of Developmental Origins of Health and Disease. 2018;9(1):30-40.
178. van Heyningen T, Honikman S, Tomlinson M, Field S, Myer L. Comparison of mental health screening tools for detecting antenatal depression and anxiety disorders in South African women. PLoS ONE [Electronic Resource]. 2018;13(4):e0193697.
179. Koen N, Brittain K, Donald KA, Barnett W, Koopowitz S, Mare K, et al. Maternal Posttraumatic Stress Disorder and Infant Developmental Outcomes in a South African Birth Cohort Study. Psychological Trauma-Theory Research Practice and Policy. 2017;9(3):292-300.
180. van Heyningen T, Honikman S, Myer L, Onah MN, Field S, Tomlinson M. Prevalence and predictors of anxiety disorders amongst low-income pregnant women in urban South Africa: a cross-sectional study. Archives of Women's Mental Health. 2017;20(6):765-75.
181. Choi KW, Sikkema KJ, Velloza J, Marais A, Jose C, Stein DJ, et al. Maladaptive coping mediates the influence of childhood trauma on depression and PTSD among pregnant women in South Africa. Archives of Women's Mental Health. 2015;18(5):731-8.
182. Patabendige M, Wanniarachchi D, Weerasinghe M, Ruwanpathirana P, Jayasundara D, Jayawardane A. The sustained adverse impact of COVID-19 pandemic on mental health among pregnant women in Sri Lanka: a reassessment during the second wave. BMC research notes. 2022;15(1):3.
183. Gankanda WI, Gunathilake IAGMP, Kahawala NL, Ranaweera AKP. Prevalence and associated factors of post-traumatic stress disorder (PTSD) among a cohort of Srilankan post-partum mothers: a cross-sectional study. BMC Pregnancy and Childbirth. 2021;21(1) (no pagination).
184. Patabendige M, Gamage MM, Weerasinghe M, Jayawardane A. Psychological impact of the COVID-19 pandemic among pregnant women in Sri Lanka. International Journal of Gynecology & Obstetrics. 2020;151(1):150-3.

185. Priyadarshanie MN, Waas MDIA, Goonewardena CSE, Balasuriya A, Senaratna BCV, Fernando DMS. Sinhala translation of the Perinatal Anxiety Screening Scale: A valid and reliable tool to detect anxiety disorders among antenatal women. BMC Psychiatry. 2020;20(1).
186. Ngocho JS, Watt MH, Minja L, Knettel BA, Mmbaga BT, Williams P, et al. Depression and anxiety among pregnant women living with HIV in Kilimanjaro region, Tanzania. PLoS ONE. 2019;14(10).
187. Mahenge B, Stockl H, Likindikoki S, Kaaya S, Mbwambo J. The prevalence of mental health morbidity and its associated factors among women attending a prenatal clinic in Tanzania. International Journal of Gynecology & Obstetrics. 2015;130(3):261-5.
188. Mahenge B, Likindikoki S, Stockl H, Mbwambo J. Intimate partner violence during pregnancy and associated mental health symptoms among pregnant women in Tanzania: a cross-sectional study. BJOG: An International Journal of Obstetrics & Gynaecology. 2013;120(8):940-6.
189. Rees SJ, Tol W, Mohammad M, Tay AK, Tam N, dos Reis N, et al. A high-risk group of pregnant women with elevated levels of conflict-related trauma, intimate partner violence, symptoms of depression and other forms of mental distress in post-conflict Timor-Leste. Translational Psychiatry. 2016;6.
190. Silove D, Rees S, Tay AK, da Costa ZM, Savio ES, Soares C, et al. Pathways to perinatal depressive symptoms after mass conflict in Timor-Leste: a modelling analysis using cross-sectional data. The Lancet Psychiatry. 2015;2(2):161-7.
191. Caglayan Keles N. The risk of anxiety and depression among pregnant women during the COVID-19 pandemic in Turkey: A cross-sectional online survey. African journal of reproductive health. 2023;27(4):65-72.
192. Aba YA, Dulger O, Sik BA, Ozolcay O. Levels and Predictors of Anxiety and Depression in Turkish Pregnant Woman During the Covid-19 Pandemic. Revista Brasileira de Ginecologia e Obstetricia.44(2):100-8.
193. Cankaya S, Ibrahimoglu T. Stress, anxiety, intolerance of uncertainty, and psychological well-being characteristics of pregnant women with and without threatened miscarriage: a case-control study. Journal of Obstetrics and Gynaecology. 2022;42(8):3577-83.
194. Keskin DD, Keskin S, Bostan S. Mental disorders among pregnant women during the COVID-19 pandemic. A cross-sectional study. Sao Paulo Medical Journal. 2022;140(1):87-93.
195. Koyucu RG, Karaca PP. The Covid 19 outbreak: Maternal Mental Health and Associated Factors. Midwifery. 2021;99:103013.
196. Ayaz R, Hocaoglu M, Gunay T, Yardimci OD, Turgut A, Karateke A. Anxiety and depression symptoms in the same pregnant women before and during the COVID-19 pandemic. Journal of Perinatal Medicine. 2020;48(9):965-70.
197. Sut HK, Kucukkaya B. Anxiety, depression, and related factors in pregnant women during the COVID-19 pandemic in Turkey: A web-based cross-sectional study. Perspectives in Psychiatric Care. 2020.
198. Yassa M, Yassa A, Yirmibes C, Birol P, Unlu UG, Tekin AB, et al. Anxiety levels and obsessive compulsion symptoms of pregnant women during the COVID-19 pandemic. Turkish Journal of Obstetrics and Gynecology. 2020;17(3):155-60.
199. Dikmen-Yildiz P, Ayers S, Phillips L. Factors associated with post-traumatic stress symptoms (PTSS) 4-6 weeks and 6 months after birth: A longitudinal population-based study. Journal of Affective Disorders. 2017;221:238-45.
200. Dikmen-Yildiz P, Ayers S, Phillips L. Depression, anxiety, PTSD and comorbidity in perinatal women in Turkey: A longitudinal population-based study. Midwifery. 2017;55:29-37.
201. Nampijja M, Natamba B, Mpango R, Kinyanda E. The burden and risk factors for postnatal depression and depressive symptomatology among women in Kampala. Tropical Doctor. 2019;49(3):170-7.
202. Luong TC, Pham TTM, Nguyen MH, Do AQ, Pham LV, Nguyen HC, et al. Fear, anxiety and depression among pregnant women during COVID-19 pandemic: impacts of healthy eating behaviour and health literacy. Annals of Medicine. 2021;53(1):2120-31.
203. Fisher J, Tran T, La BT, Kriitmaa K, Rosenthal D. Common perinatal mental disorders in northern Viet Nam: community prevalence and health care use. Bulletin of the World Health Organization. 2010;88(10):737-45.

© 2023 Roddy Mitchell A et al. *JAMA Network Open.*

eFigure 1: Forest plot anxiety disorder

Study	Number of successes	Total	No. of successes per 100 obs with 95% CI	Weight (%)
Begum	723	1,360	53.16 [50.51, 55.81]	0.52
Nasreen	212	720	29.44 [26.17, 32.83]	0.52
Edhborg	234	671	34.87 [31.31, 38.52]	0.52
Nasreen	149	583	25.56 [22.09, 29.18]	0.52
Dias	15	94	15.96 [9.17, 24.12]	0.50
Takahasi	591	1,447	40.84 [38.32, 43.39]	0.52
Pinheiro	72	828	8.70 [6.87, 10.72]	0.52
Faisal-Cury	257	432	59.49 [54.82, 64.08]	0.52
MônicaMariadeJesus	56	209	26.79 [20.99, 33.02]	0.52
Scholl	227	1,852	12.26 [10.80, 13.79]	0.52
Silva	29	260	11.15 [7.59, 15.29]	0.52
deMello	107	980	10.92 [9.04, 12.95]	0.52
Costa	58	300	19.33 [15.05, 24.01]	0.52
Lopes	547	1,279	42.77 [40.07, 45.49]	0.52
Farias	87	188	46.28 [39.18, 53.45]	0.51
Coelho	72	828	8.70 [6.87, 10.72]	0.52
Soares	59	645	9.15 [7.04, 11.50]	0.52
Muller	25	250	10.00 [6.56, 14.05]	0.52
CastroeCouto	9	148	6.08 [2.71, 10.58]	0.51
Ferraro	126	775	16.26 [13.74, 18.94]	0.52
Farias	25	239	10.46 [6.87, 14.69]	0.52
Pabon	159	531	29.94 [26.12, 33.91]	0.52
Peter	76	871	8.73 [6.94, 10.70]	0.52
Tavares	113	919	12.30 [10.25, 14.50]	0.52
Paskulin	141	712	19.80 [16.95, 22.81]	0.52
Nomura	762	1,662	45.85 [43.46, 48.25]	0.52
deMatos	74	870	8.51 [6.74, 10.46]	0.52
Loret de Mola	246	2,307	10.66 [9.44, 11.96]	0.52
daSilva	460	1,334	34.48 [31.95, 37.06]	0.52
Jiang	339	1,873	18.10 [16.39, 19.88]	0.52
Wang	1,091	15,428	7.07 [6.67, 7.48]	0.53
Pages	4	484	0.83 [0.18, 1.87]	0.52

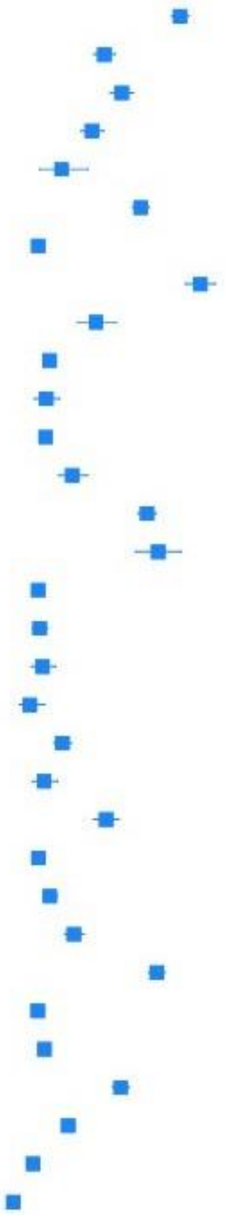

© 2023 Roddy Mitchell A et al. *JAMA Network Open.*

Liu	334	1,947	17.15 [15.51, 18.86]	0.52
Jiang	71	309	22.98 [18.45, 27.84]	0.52
Zeng	195	625	31.20 [27.62, 34.89]	0.52
Duan	41	1,027	3.99 [2.87, 5.28]	0.52
Luo	255	2,140	11.92 [10.58, 13.32]	0.52
Tang	182	1,220	14.92 [12.97, 16.97]	0.52
Zhou	287	4,890	5.87 [5.23, 6.55]	0.53
Zhang	11	365	3.01 [1.47, 5.05]	0.52
Dong	26	156	16.67 [11.19, 22.96]	0.51
Ma	258	1,583	16.30 [14.52, 18.16]	0.52
Zhou	9	210	4.29 [1.90, 7.51]	0.52
Yang	1,519	19,515	7.78 [7.41, 8.16]	0.53
Zeng	54	156	34.62 [27.33, 42.28]	0.51
Wang	37	681	5.43 [3.85, 7.27]	0.52
Liu	22	205	10.73 [6.83, 15.37]	0.51
Ma	242	665	36.39 [32.77, 40.09]	0.52
Shi	279	2,979	9.37 [8.34, 10.44]	0.53
Xie	306	2,657	11.52 [10.33, 12.76]	0.53
Wu	337	3,434	9.81 [8.84, 10.83]	0.53
Kang	96	467	20.56 [17.01, 24.35]	0.52
Yang	151	1,963	7.69 [6.55, 8.91]	0.52
Liu	183	1,204	15.20 [13.23, 17.28]	0.52
Cao	3	42	7.14 [0.93, 17.32]	0.48
Zhang	448	2,150	20.84 [19.15, 22.58]	0.52
Yue	6	308	1.95 [0.65, 3.85]	0.52
Ding	170	817	20.81 [18.09, 23.66]	0.52
Ge	51	446	11.43 [8.64, 14.56]	0.52
Gao	19	916	2.07 [1.24, 3.11]	0.52
Zhang	143	778	18.38 [15.73, 21.18]	0.52
Lin	101	751	13.45 [11.10, 15.99]	0.52
Chen	101	828	12.20 [10.05, 14.52]	0.52
Guo	252	5,728	4.40 [3.88, 4.95]	0.53
Jiang	115	718	16.02 [13.42, 18.79]	0.52
Yu	445	2,115	21.04 [19.33, 22.80]	0.52
Zhang	1	113	0.88 [0.68, 3.76]	0.51

© 2023 Roddy Mitchell A et al. *JAMA Network Open.*

Jigeer	152	2,018	7.53 [6.42, 8.73]	0.52
Xie	112	689	16.26 [13.59, 19.11]	0.52
Chen	374	1,338	27.95 [25.58, 30.39]	0.52
Yu	64	813	7.87 [6.11, 9.83]	0.52
Qiao	36	527	6.83 [4.82, 9.15]	0.52
Mo	428	4,087	10.47 [9.55, 11.43]	0.53
Hou	117	757	15.46 [12.96, 18.12]	0.52
Zhang	620	1,794	34.56 [32.38, 36.78]	0.52
Xu	38	274	13.87 [10.01, 18.23]	0.52
Yang	114	717	15.90 [13.31, 18.67]	0.52
Ma	311	2,813	11.06 [9.92, 12.24]	0.53
Luo	17	97	17.53 [10.54, 25.79]	0.50
Peng	3	71	4.23 [0.54, 10.43]	0.50
Qin	10	169	5.92 [2.79, 10.05]	0.51
Cui	34	304	11.18 [7.87, 14.99]	0.52
Zhou	37	544	6.80 [4.83, 9.08]	0.52
Yang	140	770	18.18 [15.53, 20.99]	0.52
Yang	19	121	15.70 [9.71, 22.78]	0.51
Cao	15	256	5.86 [3.27, 9.11]	0.52
Lu	99	3,110	3.18 [2.59, 3.83]	0.53
Wassif	156	500	31.20 [27.21, 35.34]	0.52
Abdelhai	43	376	11.44 [8.40, 14.86]	0.52
Ahmed	153	238	64.29 [58.08, 70.26]	0.52
Bante	95	667	14.24 [11.69, 17.00]	0.52
Tibebu	178	423	42.08 [37.41, 46.82]	0.52
Malaju	143	775	18.45 [15.80, 21.26]	0.52
Beketie	73	316	23.10 [18.61, 27.92]	0.52
Kassaw	57	178	32.02 [25.35, 39.08]	0.51
Bishaw	352	806	43.67 [40.26, 47.11]	0.52
Lelisho	118	423	27.90 [23.72, 32.27]	0.52
Kugbey	76	214	35.51 [29.23, 42.06]	0.52
Ray	31	124	25.00 [17.74, 33.03]	0.51
Boggaram	12	100	12.00 [6.27, 19.18]	0.50
Tikka	69	620	11.13 [8.77, 13.73]	0.52
Jha	48	457	10.50 [7.85, 13.49]	0.52

© 2023 Roddy Mitchell A et al. *JAMA Network Open.*

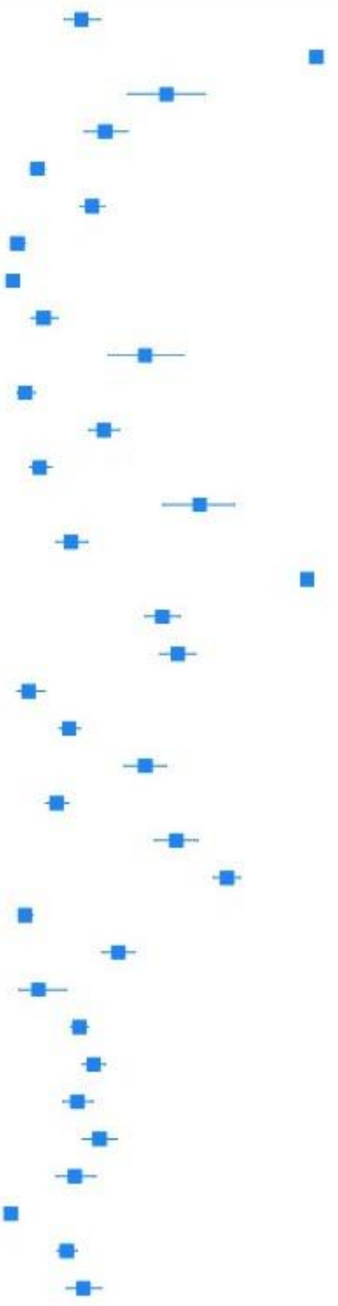

Kantipudi	49	209	23.44 [17.93, 29.45]	0.52
Thurkkada	380	384	98.96 [97.64, 99.78]	0.52
Nazir	32	63	50.79 [38.41, 63.13]	0.49
Tiwari	56	180	31.11 [24.54, 38.08]	0.51
Ture	60	650	9.23 [7.12, 11.58]	0.52
Bhushan	129	480	26.88 [23.00, 30.94]	0.52
Ryali	7	245	2.86 [1.08, 5.38]	0.52
Goyal	4	281	1.42 [0.30, 3.22]	0.52
Maria	26	231	11.26 [7.47, 15.68]	0.52
Khatri	29	66	43.94 [32.12, 56.11]	0.49
Bachani	13	243	5.35 [2.83, 8.58]	0.52
Jelly	102	333	30.63 [25.79, 35.70]	0.52
Singh	28	280	10.00 [6.74, 13.81]	0.52
Zainiyah	43	70	61.43 [49.69, 72.55]	0.50
Margawati	48	238	20.17 [15.30, 25.52]	0.52
Maleki	2,243	2,336	96.02 [95.19, 96.78]	0.52
Keramat	146	295	49.49 [43.79, 55.20]	0.52
Alipour	161	296	54.39 [48.69, 60.04]	0.52
Mahmoodi	8	124	6.45 [2.70, 11.55]	0.51
Maharlouei	105	540	19.44 [16.21, 22.90]	0.52
Effati-Daryani	90	205	43.90 [37.16, 50.76]	0.51
Youseflu	59	380	15.53 [12.05, 19.35]	0.52
Basha	108	200	54.00 [47.05, 60.87]	0.51
Gerges	304	433	70.21 [65.81, 74.43]	0.52
Kolak	22	415	5.30 [3.33, 7.68]	0.52
Jusoh	113	320	35.31 [30.16, 40.64]	0.52
Azlan	6	62	9.68 [3.35, 18.48]	0.49
Nasreen	206	904	22.79 [20.11, 25.58]	0.52
Nasreen	155	566	27.39 [23.79, 31.14]	0.52
Padilla	62	280	22.14 [17.46, 27.21]	0.52
Hababa	74	253	29.25 [23.79, 35.02]	0.52
Aryal	35	164	21.34 [15.38, 27.97]	0.51
Shrestha	2	273	0.73 [0.01, 2.20]	0.52
Aryal	106	567	18.69 [15.59, 22.01]	0.52
Shrestha	52	216	24.07 [18.59, 30.02]	0.52

© 2023 Roddy Mitchell A et al. *JAMA Network Open.*

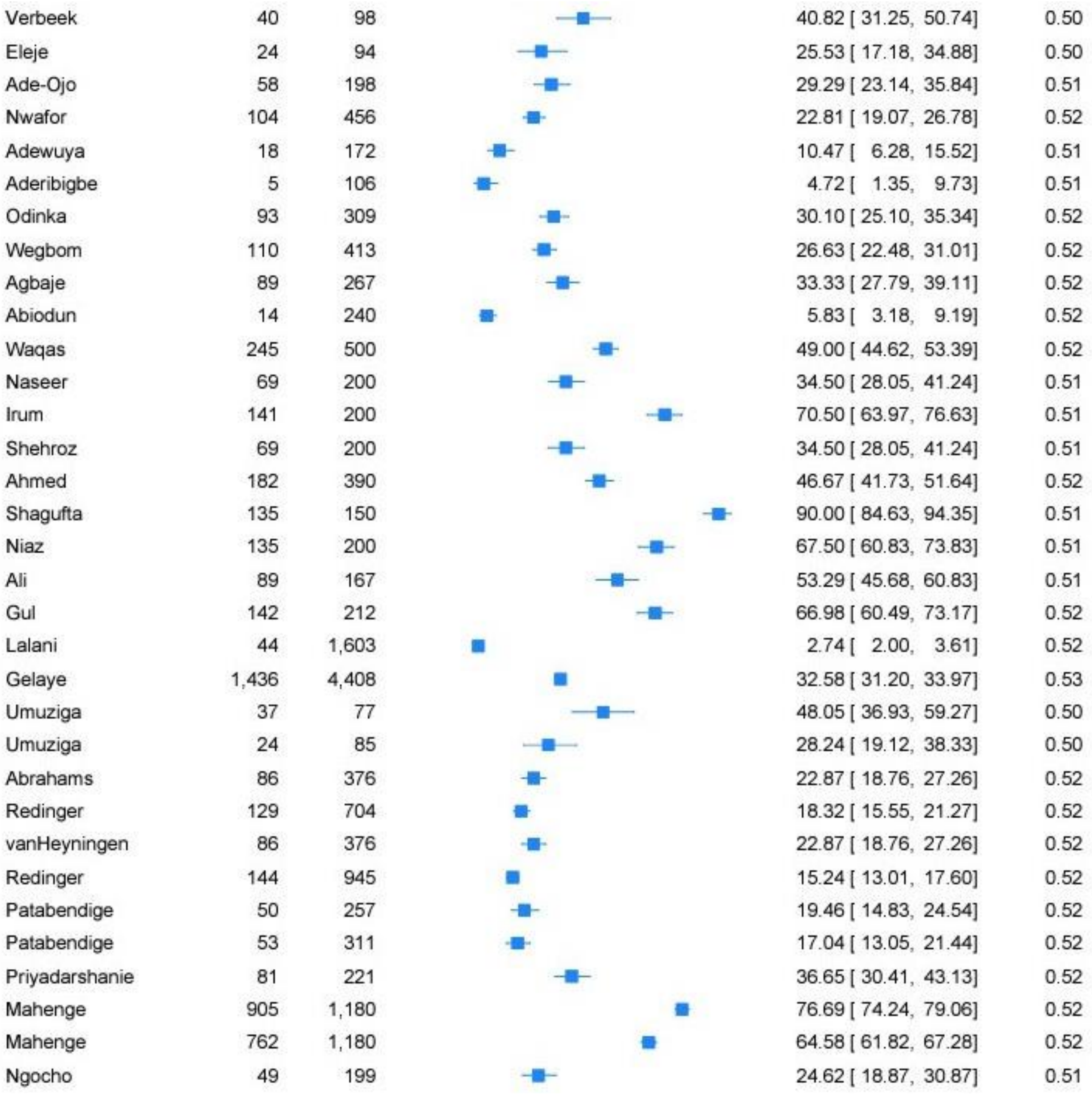

© 2023 Roddy Mitchell A et al. *JAMA Network Open.*

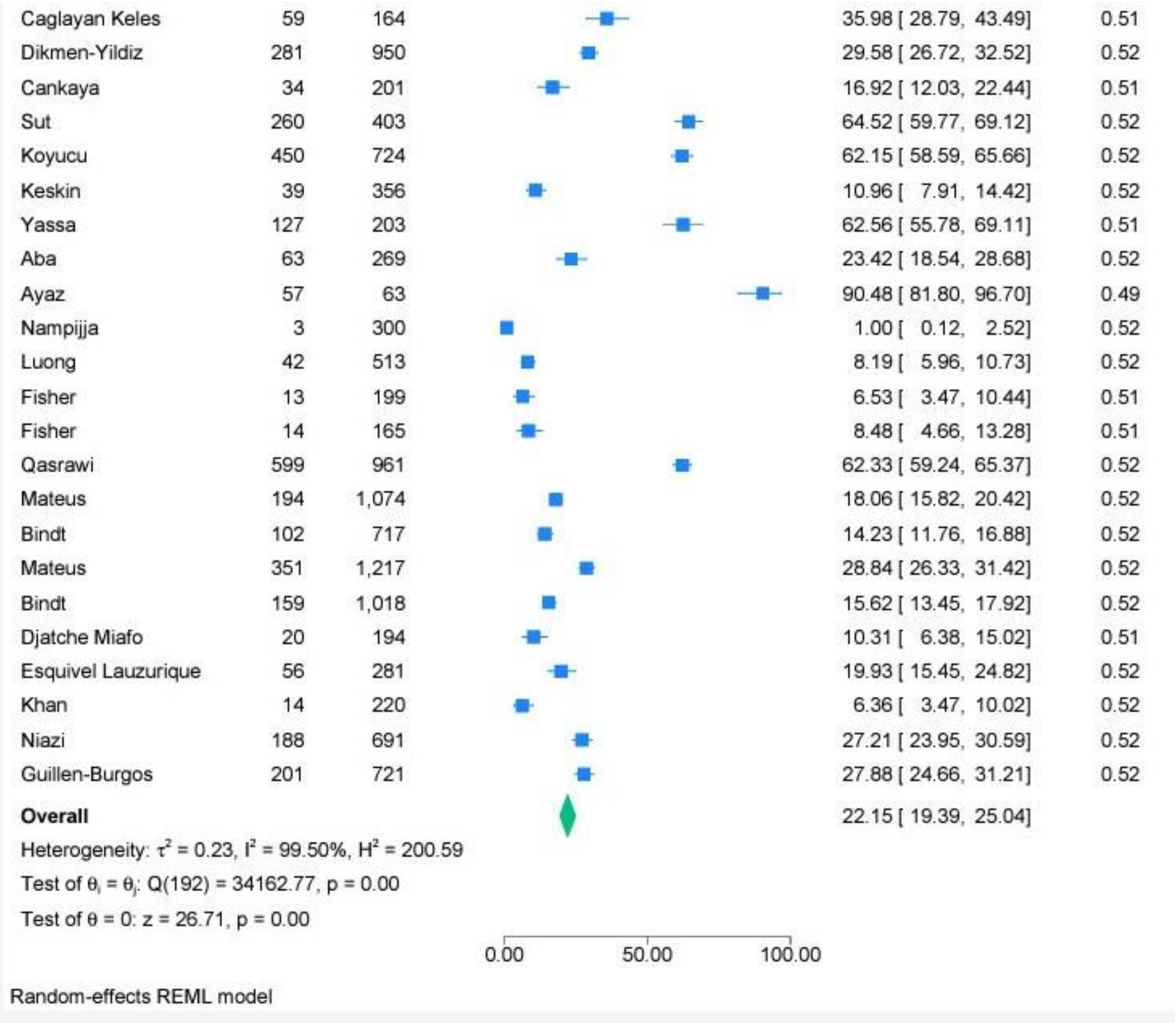

© 2023 Roddy Mitchell A et al. *JAMA Network Open.*

eFigure 2: Forest plot post-traumatic stress disorder

Study	Number of successes	Total	No. of successes per 100 obs with 95% CI	Weight (%)
Farias	4	239	1.67 [0.36, 3.78]	3.01
Pinheiro	21	828	2.54 [1.56, 3.73]	3.07
Coelho	21	828	2.54 [1.56, 3.73]	3.07
Tavares	33	919	3.59 [2.48, 4.90]	3.07
CastroeCouto	1	148	0.68 [0.52, 2.88]	2.95
Silva	8	260	3.08 [1.27, 5.58]	3.01
Zambaldi	21	400	5.25 [3.26, 7.67]	3.04
Peter	21	871	2.41 [1.49, 3.55]	3.07
Zhang	752	1,901	39.56 [37.37, 41.77]	3.08
Qu	38	311	12.22 [8.80, 16.11]	3.03
Qu	63	317	19.87 [15.65, 24.46]	3.03
Zhou	5	544	0.92 [0.26, 1.93]	3.06
Ahmed	13	238	5.46 [2.89, 8.75]	3.01
Malaju	75	775	9.68 [7.69, 11.86]	3.07
Ryali	3	245	1.22 [0.15, 3.08]	3.01
Mahmoodi	15	240	6.25 [3.50, 9.70]	3.01
Shaban	103	600	17.17 [14.25, 20.29]	3.06
Bataineh	282	481	58.63 [54.19, 63.00]	3.05
Adewuya	1	172	0.58 [0.45, 2.48]	2.97
Gelaye	1,519	4,408	34.46 [33.06, 35.87]	3.09
Mare	61	347	17.58 [13.74, 21.77]	3.03
Koen	22	111	19.82 [12.88, 27.80]	2.91
vanHeyningen	41	376	10.90 [7.94, 14.27]	3.04
Choi	26	84	30.95 [21.47, 41.30]	2.86
Gankanda	6	225	2.67 [0.89, 5.25]	3.00
Mahenge	58	1,180	4.92 [3.75, 6.23]	3.08
Mahenge	150	1,180	12.71 [10.87, 14.68]	3.08
Silove	40	387	10.34 [7.49, 13.58]	3.04
Rees	96	1,672	5.74 [4.67, 6.91]	3.08
Dikmen-Yildiz	55	950	5.79 [4.39, 7.37]	3.07
Nampijja	1	300	0.33 [0.26, 1.43]	3.02
Djatche Miafo	6	194	3.09 [1.04, 6.08]	2.99
Guillen-Burgos	20	721	2.77 [1.69, 4.11]	3.06
**Overall**			8.28 [5.03, 12.22]	

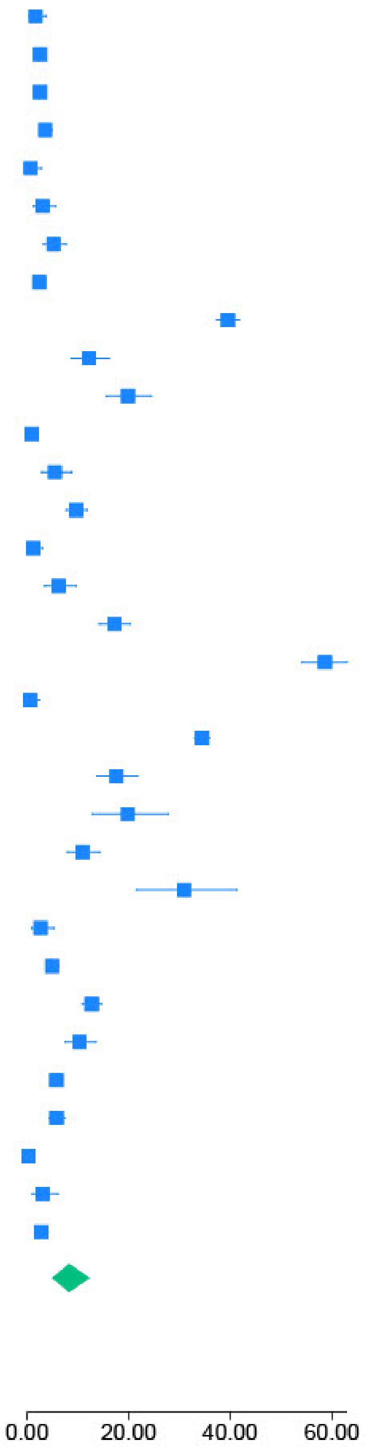

Heterogeneity: $\tau^2 = 0.14$, $I^2 = 98.92\%$, $H^2 = 92.89$

Test of $\theta_i = \theta_j$: Q(32) = 4026.58, p = 0.00

Test of $\theta = 0$: z = 8.07, p = 0.00

Random-effects REML model

© 2023 Roddy Mitchell A et al. *JAMA Network Open.*

eFigure 3: Forest plot obsessive-compulsive disorder

Study	Number of successes	Total	No. of successes per 100 obs with 95% CI	Weight (%)
Tavares	48	919	5.22 [3.87, 6.76]	5.95
Pinheiro	29	828	3.50 [2.35, 4.87]	5.95
Silva	9	260	3.46 [1.53, 6.08]	5.86
Farias	8	239	3.35 [1.38, 6.06]	5.85
Ferraro	35	775	4.52 [3.16, 6.10]	5.95
Zambaldi	36	400	9.00 [6.38, 12.02]	5.91
Coelho	29	828	3.50 [2.35, 4.87]	5.95
Peter	32	871	3.67 [2.52, 5.03]	5.95
CastroeCouto	3	148	2.03 [0.25, 5.08]	5.77
Liu	36	205	17.56 [12.63, 23.09]	5.83
Ture	3	650	0.46 [0.06, 1.17]	5.94
Ryali	3	245	1.22 [0.15, 3.08]	5.86
Goyal	1	281	0.36 [0.27, 1.52]	5.87
Adewuya	9	172	5.23 [2.33, 9.13]	5.80
Malemela	81	206	39.32 [32.74, 46.09]	5.83
vanHeyningen	15	376	3.99 [2.22, 6.23]	5.90
Yassa	125	203	61.58 [54.77, 68.16]	5.83
**Overall**			6.91 [2.60, 13.02]	

Heterogeneity: $\tau^2 = 0.18$, $I^2 = 98.76\%$, $H^2 = 80.34$

Test of $\theta_i = \theta_j$: $Q(16) = 704.34$, $p = 0.00$

Test of $\theta = 0$: $z = 4.61$, $p = 0.00$

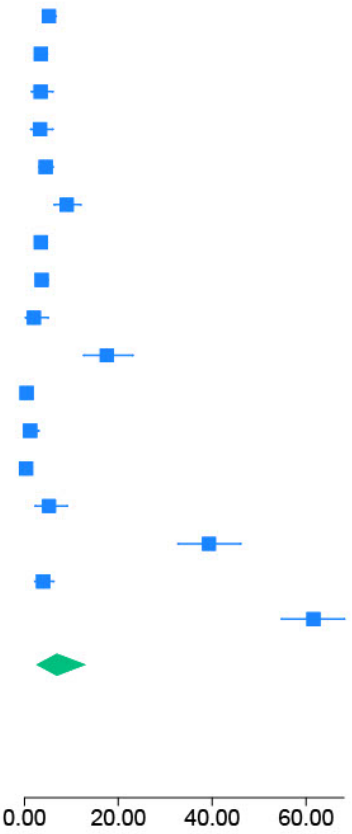

Random-effects REML model

© 2023 Roddy Mitchell A et al. *JAMA Network Open.*

eFigure 4: Forest plot panic disorder

Study	Number of successes	Total	No. of successes per 100 obs with 95% CI	Weight (%)
Coelho	18	828	2.17 [1.28, 3.29]	7.78
Pinheiro	18	828	2.17 [1.28, 3.29]	7.78
Farias	1	239	0.42 [0.32, 1.79]	7.01
Tavares	31	919	3.37 [2.29, 4.65]	7.81
Silva	3	260	1.15 [0.14, 2.90]	7.09
Peter	18	871	2.07 [1.22, 3.13]	7.79
Jha	49	457	10.72 [8.04, 13.73]	7.51
Ryali	14	245	5.71 [3.11, 9.01]	7.04
Fadzil	10	175	5.71 [2.69, 9.71]	6.68
Adewuya	9	172	5.23 [2.33, 9.13]	6.66
Tariq	23	150	15.33 [9.97, 21.58]	6.49
Fisher	3	199	1.51 [0.19, 3.79]	6.83
Fisher	7	165	4.24 [1.61, 7.94]	6.61
Khan	5	220	2.27 [0.64, 4.74]	6.93
**Overall**			3.70 [2.14, 5.63]	

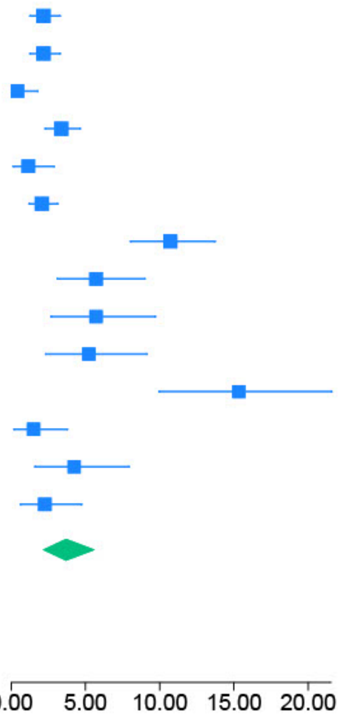

Heterogeneity: $\tau^2 = 0.03$, $I^2 = 91.14\%$, $H^2 = 11.29$

Test of $\theta_i = \theta_j$: Q(13) = 111.20, p = 0.00

Test of $\theta = 0$: z = 7.25, p = 0.00

Random-effects REML model

© 2023 Roddy Mitchell A et al. *JAMA Network Open.*

eFigure 5: Forest plot social anxiety disorder

Study	Number of successes	Total	No. of successes per 100 obs with 95% CI	Weight (%)
Tavares	45	919	4.90 [3.59, 6.39]	18.32
Silva	14	260	5.38 [2.93, 8.50]	16.69
Coelho	42	828	5.07 [3.67, 6.68]	18.24
Fadzil	1	175	0.57 [0.44, 2.44]	15.75
Adewuya	11	172	6.40 [3.16, 10.60]	15.70
Tariq	21	150	14.00 [8.86, 20.05]	15.30
**Overall**			5.33 [2.56, 8.99]	

Heterogeneity: $\tau^2 = 0.03$, $I^2 = 90.88\%$, $H^2 = 10.96$

Test of $\theta_i = \theta_j$: Q(5) = 29.20, p = 0.00

Test of $\theta = 0$: z = 5.71, p = 0.00

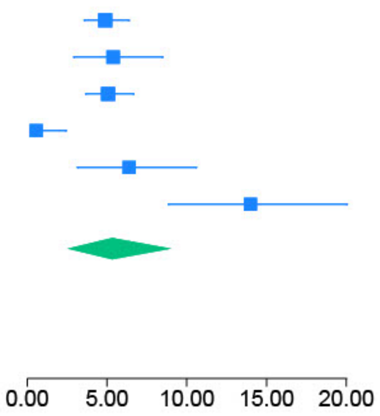

Random-effects REML model

© 2023 Roddy Mitchell A et al. *JAMA Network Open.*

eFigure 6: Forest plot adjustment disorder

Study	Number of successes	Total	No. of successes per 100 obs with 95% CI	Weight (%)
Goyal	1	281	0.36 [0.27, 1.52]	50.43
Djatche Miafo	15	194	7.73 [4.34, 11.96]	49.57
**Overall**			2.93 [0.00, 14.12]	

Heterogeneity: $\tau^2 = 0.09$, $I^2 = 95.25\%$, $H^2 = 21.04$

Test of $\theta_i = \theta_j$: Q(1) = 21.04, p = 0.00

Test of $\theta = 0$: z = 1.36, p = 0.18

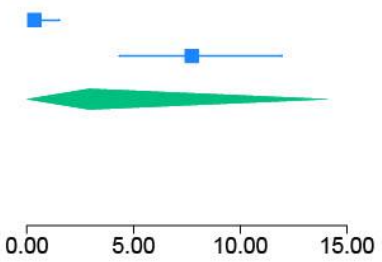

Random-effects REML model

© 2023 Roddy Mitchell A et al. *JAMA Network Open.*

eFigure 7: Map - number of studies by country

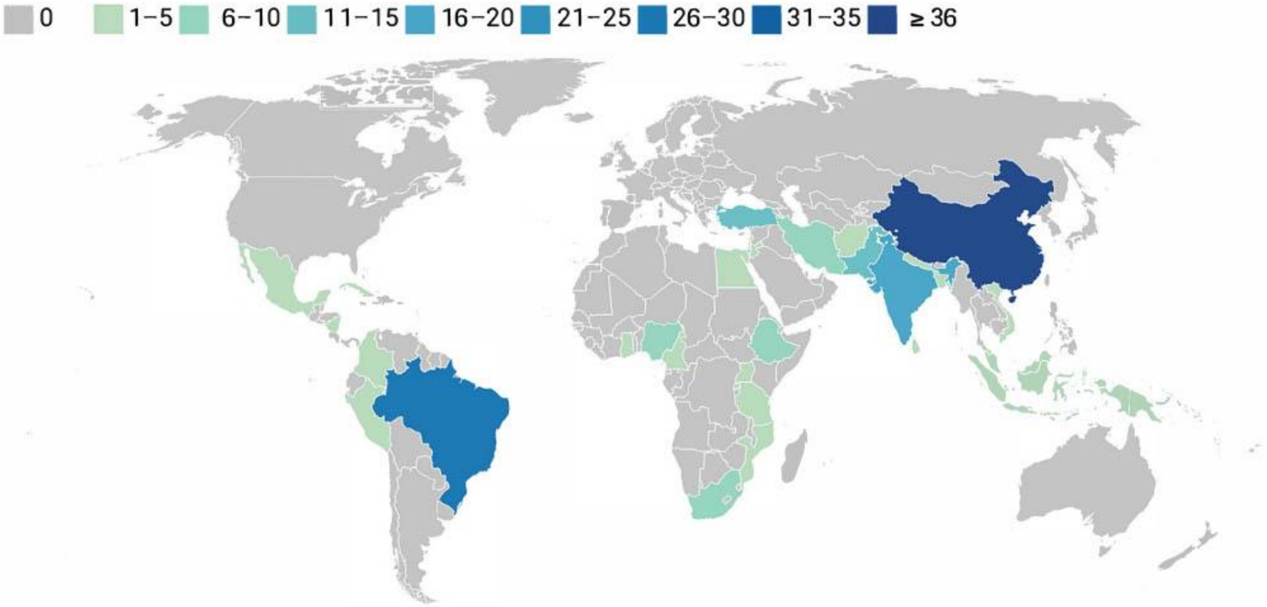

© 2023 Roddy Mitchell A et al. *JAMA Network Open.*
