# Supplementary material for: Prevalence of Perinatal Anxiety and Related Disorders in Low- and Middle-Income Countries: A Systematic Review and Meta-Analysis
Source: JAMA Netw Open. 2023 Nov 17;6(11):e2343711. doi: 10.1001/jamanetworkopen.2023.43711 (PMC10656650; doi:10.1001/jamanetworkopen.2023.43711)
Supplement: Supplement 2. — Data Sharing Statement [file jamanetwopen-e2343711-s002.pdf]

Data Sharing Statement

Roddy Mitchell. Prevalence of Perinatal Anxiety and Related Disorders in Low- and Middle-Income Countries. *JAMA Netw Open*. Published November 17, 2023. doi:10.1001/jamanetworkopen.2023.43711

Data
Data available: No

Additional Information
Explanation for why data not available: Data will be made available upon reasonable request.
